# Bi-modal confirmation of liposome delivery to the brain after focused ultrasound-induced blood-brain barrier opening

**DOI:** 10.1016/j.heliyon.2024.e39972

**Published:** 2024-11-06

**Authors:** Chris Payne, Paul Cressey, Anisia Talianu, Elwira Szychot, Darren Hargrave, Maya Thanou, Antonios N. Pouliopoulos

**Affiliations:** a School of Biomedical Engineering & Imaging Sciences, King's College London, London, UK; b Institute of Pharmaceutical Science, School of Cancer and Pharmaceutical Sciences, King's College London, London, UK; c Great Ormond Street Hospital for Children, London, UK; d Department of Developmental Biology & Cancer, University College London, London, UK; e Pomeranian Medical University, Szczecin, Poland

## Abstract

Focused ultrasound-mediated opening of the blood-brain barrier offers a great opportunity to deliver therapeutics into hard-to-treat brain tumors such as glioblastoma multiforme or diffuse midline glioma. However, the potential of the technique to offer a time window for efficient nanomedicine delivery has not been thoroughly studied. Non-invasive and targeted delivery of large drug-loaded nanocarriers, such as liposomes, could offer a safe and scalable method of personalized therapy for the treatment of brain pathologies. Additionally, it is essential to monitor the safety and efficacy of such treatments, tracking drug delivery in real-time through quantitative medical imaging.

In this study, liposomes were modified to have an MRI contrast agent (i.e., Gd) in both lipid membrane and core, while an infrared dye (i.e., CW800) was coupled to lipids introduced in the lipid bilayer for bimodal detection and treatment verification. Targeted delivery of 110 nm-in-diameter liposomes to the brain was quantified using 9.4-T MRI and near infrared fluorescence imaging. The spatiotemporal distribution of liposomes *in vivo* was assessed up to 4 h post treatment using T_1_ weighted MRI. *In vivo* MRI signal co-localized with NIRF signal from excised brains *ex vivo*. Passive acoustic detection during treatments revealed a correlation between acoustic signal and MRI contrast, providing a scalable metric for assessing clinical treatment efficacy in real-time. In conclusion, therapeutic ultrasound exposure can enhance delivery of large trackable nanoparticles into the brain, while enabling real-time treatment monitoring and verification.

## Highlights

• Focused ultrasound in conjunction with microbubbles allows blood-brain barrier opening in a targeted and non-invasive manner.
• Liposomes are versatile drug carriers that can be delivered to the brain using clinically relevant therapeutic ultrasound.
• Imageable liposomes can be tracked using MRI and near infrared fluorescence imaging, confirming their *in vivo* biodistribution.
• Passive acoustic measurements correlate with liposome delivery *in vivo*, providing a scalable method for therapy confirmation.

## Graphical abstract

**Figure:**
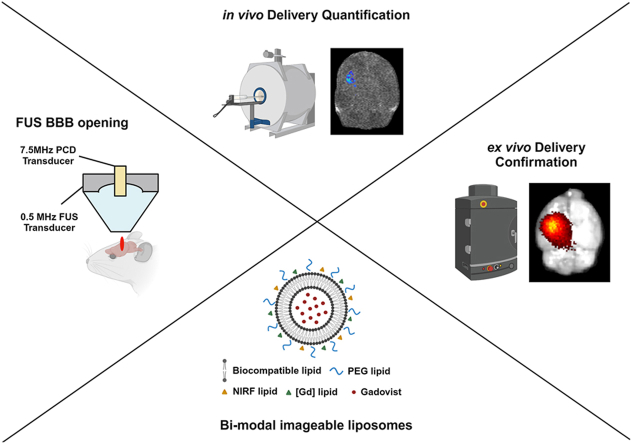

## 1 Introduction

Malignant brain tumors such as glioblastoma multiforme (GBM) and diffuse midline glioma (DMG) have very poor prognosis and universal fatality, given the limited treatment options. Discovery of promising drugs for treatment of these tumors remains challenging. This, in part, is due to the presence of the blood-brain barrier (BBB), which prevents the delivery of therapeutic concentration of drugs [1]. Focused ultrasound (FUS) in conjunction with systemically administered microbubbles is an emerging technology that can reversibly open the BBB to enable delivery of chemotherapeutics to brain tumors in a non-invasive manner while avoiding healthy tissue.

The technique uses microbubbles (1–10 μm diameter) that consist of lipid, polymer or protein shell encapsulating a gas core [[2], [3], [4]]. When subjected to ultrasound, the compression and rarefaction phases of the pressure field cause them to volumetrically oscillate, resulting in targeted BBB disruption, thereby enhancing barrier permeability to allow drug uptake into the brain. *In vivo* studies have demonstrated that various small molecule drugs, antibodies, viral vectors, and nanoparticles can be delivered into the brain using this technique [[5], [6], [7]]. The safety of FUS-induced BBB opening has been reported and established in a multitude of pre-clinical and clinical studies [[8], [9], [10], [11], [12], [13], [14]].

At present, acoustic cavitation signals in the form of microbubble acoustic emissions are typically monitored in real-time using either passive cavitation detection (PCD) or passive acoustic mapping (PAM) to ensure safety thresholds are not exceeded [10,15]. However, the ability to accurately control and alter cavitation parameters during the procedure remains elusive [16]. There is therefore a need to understand the effects of cavitation activity, and its correlation to drug delivery to develop parameters that lead to controllable and safe BBB opening.

Liposomes are commonly used nanomedicines, which have been utilized clinically to limit side effects while maintaining efficacy [[17], [18], [19]]. One main advantage of liposomes compared to other nanomedicine delivery vehicles is their highly adaptable formulation allowing for multifunctional properties, including high drug loading, introduction of contrast enhancement for clinical imaging modalities (theranostics), and triggered drug release. For example, thermosensitive liposomes can selectively release their cargo following localized non-invasive mild hyperthermia [20]. While liposomes have become a potent tool for treatment of various malignancies including breast [21] and pancreatic [22] cancers, their use in brain cancer is currently limited due to poor penetration across the BBB. Their use for brain malignancies in combination with FUS would benefit treatments by limiting systemic adverse effects and delivering substantial therapeutic doses. There are few studies that have investigated FUS-induced BBB opening to increase liposome brain uptake in rodents [[23], [24], [25]]. While liposomes have not been used clinically in the treatment of GBM or DMG to date, their ability to incorporate imaging agents allows for image-guided drug dosing and direct drug delivery confirmation, which is critical for successful treatment of any brain malignancy.

MRI is the gold standard for diagnosis of brain tumors due to its superior soft tissue contrast [26,27], while contrast-enhanced MRI has been used in the clinic to confirm BBB opening [28], using gadolinium-based contrast agents as a surrogate for drugs. Using a nanomedicine that provides MRI contrast is therefore highly desirable to enable direct quantification of the nanomedicine and drug delivery, and assess its distribution in the brain over time. One proof of concept study has shown that 70–85 nm and 130–150 nm diameter liposomes can be delivered to the brain after FUS induced BBBO and imaged using a 7-T MRI scanner [24]. However, only 2 out of 7 mice receiving 130–150 nm had detectable MRI signal. Another study has shown that 100 nm fluorescent PEGylated liposomes can be successfully delivered to the brain using rapid short pulses (RaSP) and detected through post-mortem fluorescence imaging in brain slices [25].

Previous studies have employed alternative imaging modalities such as photoacoustics and color Doppler ultrasound imaging [29] or fluorescence imaging based on labelled microbubbles [30] to evaluate BBB opening. However, there has been no study that employed imageable drug carries like liposomes and confirm their delivery and biodistribution within the brain using clinically relevant treatment and imaging parameters. There is a pressing need to develop techniques that confirm not only BBB opening but also drug delivery.

Despite the developments in FUS-mediated delivery of liposomes in the brain, there are still challenges in translation of this novel therapy into the clinic. First, the liposome formulation and MR imaging sequences have not been optimized to yield sufficient signal-to-noise ratio (SNR) for scalable confirmation of Gd-labelled liposomal delivery into the brain. Second, the treatment protocol has to resemble therapeutic protocols currently applied in clinical trials. Third, the liposome kinetics within the brain parenchyma following successful delivery is currently unknown. Finally, there has been no study assessing the ability of passive cavitation measurements to predict liposome delivery.

In this study, we sought to design, characterize, and deliver 110-nm liposomes into the brain of wild-type mice through FUS-induced BBB opening. These liposomes have been previously shown to have a significant therapeutic effect in subcutaneous breast cancer mouse models [31]. However, there has been no evidence of successful delivery into the brain and confirmation through translational imaging modalities, such as MRI. MRI was used to quantify and evaluate the spatiotemporal distribution of liposomes within treated brain regions at 2- and 4-h post-treatment. Our aim was to optimize liposome constituents and MRI imaging parameters to improve detection of contrast enhancement *in vivo*. Near infrared fluorescence imaging (NIRF) was performed *ex vivo* for bimodal confirmation of liposome uptake in the brain post FUS. Additionally, we hypothesized that passive cavitation detection metrics correlate with MRI contrast enhancement and liposome delivery in the brain. The ability to provide quantitative and scalable MRI-based confirmation of liposome delivery, coupled with passive acoustic measurements, will provide greater understanding of drug delivery efficiency and spatiotemporal distribution.

## 2 Methods

### 2.1 Imageable lipid synthesis

Gadolinium (III).DOTA.DSA (MR-labelled lipid) was synthesized, purified and confirmed according to our previous report [32]. CW800.DSA was synthesized following a modification of the method reported by Centelles et al. for the synthesis of CF750.DSA. In short, DSA (4.2 mg, 7.3 μM; N, N-Distearylamidomethylamine, synthesized according to Kamaly et al. [33]), was dissolved in dry CHCl_3_ (100 μL) with distilled DIPEA (20 μL, N, N-Diisopropylethylamine). CW800.NHS was suspended in dry CHCl_3_ (200 μL) and added to the DSA/DIPEA mixture. The reaction was protected from light and stirred at room temperature for 48 h. The volatiles were removed *in vacuo* and the crude product purified by gradient silica gel column chromatography [MeOH:DCM (0:10 → 2:8 v/v). The product was confirmed by mass spectrometry (MS) and redissolved in CHCl_3_ at 1 mg/mL.

### 2.2 Liposome formulation

Thermosensitive liposomes (iTSL) were prepared using the freeze-thaw sonication method. The biocompatible lipids DPPC (1,2-Dipalmitoyl-sn-glycero-3-phosphocholine; 16:0 PC), DSPC (1,2-distearoyl-sn-glycero-3-phosphocholine; 18:0 PC), MSPC (1-stearoyl-sn-glycero-3-phosphocholine; 18:0 lyso-PC) and DSPE-PEG_2000_OMe (ω-methoxy-polyethyleneglycol2000)-N-carboxy-1,2-distearoyl-sn-glycero-3-phosphoethanolamine) were purchased from Avanti Polar Lipids (AL, USA) or Sigma Aldrich (MO, USA). All lipids were stored at −20 °C as aliquots of 25 mg/mL in chloroform. Liposomes were prepared with DPPC:DSPC:MSPC:DSPE-PEG_2000_OMe:[Gd].DOTA.DSACW800.DSA at 53.9:5:5:6:30:0.1 mol%. Lipid stocks were combined in a round bottom flask and the solvent was removed slowly *in vacuo* to ensure thin-film formation. The lipid film was then hydrated using a solution of Gadovist® (1 mM/mL, 2 mL, pH 6.6) to form liposomes in solutions with final lipid concentrations of 30 mg/mL for iTSL30[Gd] and 50 mg/mL for iTSL50[Gd]. The total lipid mass was 60 mg for iTSL30 and 100 mg for iTSL50 liposomes. The lipid film was fragmented by 10 × freeze-thaw cycles followed by sonication at 60 °C to form a homogeneous suspension of liposomes. The unencapsulated Gadovist® was removed via dialysis (100 kDa cutoff) against 20 mM HEPES buffer (pH 6.6). After purification, batches were assessed using a Nanoseries Nano ZS (Malvern Panalytical, UK). Samples were diluted 1:50 (v/v) using pH 6.6 buffer at 25 °C and size modelling used default solute and particle parameters.

### 2.3 Gadolinium quantification experiments

Gadolinium concentrations were assayed by inductively coupled plasma - optical emission spectrometry (ICP-OES) analysis using a Thermo Scientific iCAP 6300 duo and/or ICP-mass spectroscopy (ICP-MS) using a PerkinElmer NexION 350D (both Waltham, MA, USA). Samples of iTSL (10 μL) were digested overnight at 60 °C with HNO_3_ (140 μL; 68 w% aq.; Fisher Scientific) and H_2_O_2_ (50 μL; 30 w% aq.; Sigma Aldrich) in tightly sealed plastic tubes. Brain tissue samples were dried in pre-weighed plastic tubes before digestion in HNO_3_ (300 μL; 68 w% aq.; Fisher Scientific) and H_2_O_2_ (100 μL; 30 w% aq.; Sigma Aldrich). The digests were diluted 1:10 (v/v) with ultrapure water (Sigma Aldrich) and [Gd] assayed. Final concentrations for gadolinium are reported after correction for dilution and corrected to tissue weight for brain samples. Metal analysis calibration and controls used TraceCERT® certified reference materials (Sigma Aldrich).

### 2.4 MRI phantom experiments

MRI was performed on iTSL's without and with Gd encapsulation at concentrations of 30 mg/mL (iTSL30 and iTSL30 [Gd]) and 50 mg/mL with Gd encapsulated (iTSL50 [Gd]). Imaging was performed on a 9.4-T Bruker Vertical MRI scanner (Bruker, MA, USA), using a 30 mm volume coil. T_1_ weighted RARE (TR/TE = 18/600 ms, averages = 4, FOV = 20 × 20 mm, matrix size = 128 × 128, slice thickness = 1 mm), T_1_ weighted FLASH (TR/TE = 230/3.3 ms, averages = 4, FOV = 20 × 20 mm, matrix size = 128 × 128, slice thickness = 0.5 mm), T_2_ weighted TURBORARE (TR/TE = 2500/30 ms, averages = 4, FOV = 20 × 20 mm, matrix size = 128 × 128, slice thickness = 1 mm) and T_1_ mapping (T1 experiments = 8, averages = 1, FOV = 30 × 30 mm, matrix size = 128 × 128, slice thickness = 0.5 mm, echo spacing = 6.5 ms, RARE factor = 2) MRI sequences were acquired. ROI analysis was performed using Paravision within regions covering the entire MRI tube diameter to calculate average signal intensity for each sample.

### 2.5 Near infrared fluorescence (NIRF) in vitro assessment

To determine the limit of detection of the NIRF contrast lipid into the iTSL formulation NIRF imaging was performed on serially diluted iTSLs (×10, 20, 100 and 200) using an *in vivo* multispectral imaging system (IVIS™) with excitation wavelength of 745 nm, and emission wavelength of 800 nm.

### 2.6 *In vivo* FUS experiments

**Fig. 1:**
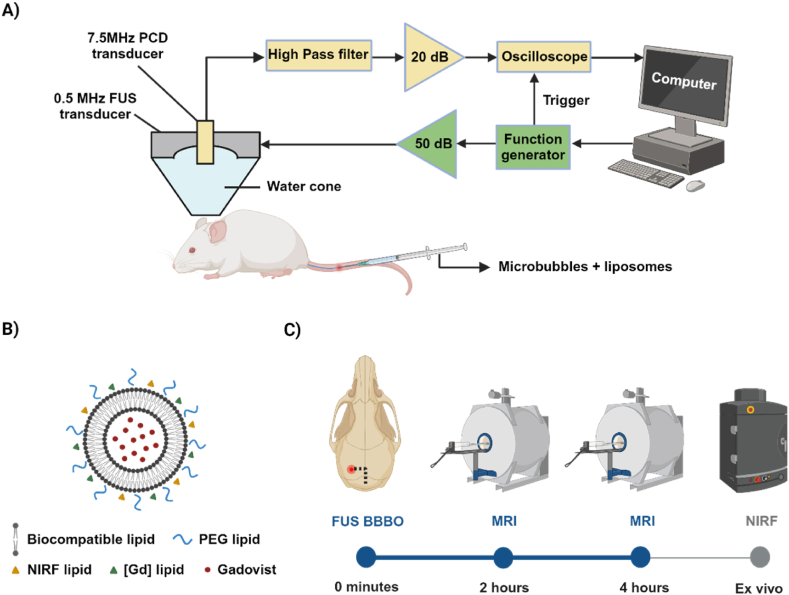
A. schematic of experimental setup. B. schematic representation of dual-labelled thermosensitive liposome. C. Timeline of *in vivo* experiments. Sonication location was +2 mm anterior/posterior, −2 mm medial/lateral from the lamboidal structure. Created with BioRender.com.

All experimental protocols were approved by the institutional animal facility committee and the UK Home Office (project license PP/5854694). *In vivo* experiments were conducted in a similar experimental setup as described in detail previously [34] (Fig. 1A). Briefly, a 0.5-MHz single-element FUS transducer (Part No. H-204; Sonic Concepts, Bothell, WA, USA) was driven by a waveform generator (33500B series; Agilent technologies, Santa Clara, CA, USA) through a radiofrequency power amplifier (A075 RF Power Amplifier, 300 kHz to 35 MHz, 75 W; E&I, Rochester, NY, USA). Acoustic emissions were captured with a 7.5-MHz single-element passive cavitation detector (Part No. U8423539, V320, diameter: 12.7 mm, focal depth: 76.2 mm; Olympus Industrial, Waltham, MA, USA) which was inserted and co-aligned with FUS transducer, having overlapping foci. A high-pass filter was used to filter out the fundamental and the second harmonic reflections (Part No. ZFHP-1R2-S+, cut-off frequency 1.2 MHz; Mini Circuits, Brooklyn, NY, USA). Recorded signals were amplified by 20-dB with a pulser-receiver (VK-71000, Gampt mbH, Merseburg, Germany) and then recorded using a Picoscope (5244D, Picotech, UK). Segments of 187,500 time points were captured at a sampling frequency of 125 MSa/s.

Experiments were performed on 7 female CD-1 mice (Charles River Laboratories, 4–6 weeks old, weight ∼ 30 g). Anesthesia was induced and maintained with inhalable isoflurane mixed with oxygen (∼4 % induction, 1.5–2% maintenance) delivery through a digital vaporizer (Somnosuite, Kent Scientific, Torrington, CT, USA). Mice were fixed in a stereotactic frame for accurate targeting. Head fur was removed with clippers and depilatory cream applied for 10–20 s. Using a metallic grid method previously described [35], the caudate area was targeted (−2 mm lateral, +2 mm ventral from lamboid suture). Caudate was targeted as it is a common tumor location. A control sonication was performed before microbubble injection to acquire a baseline signal, which was subsequently subtracted from the microbubble signal in the frequency domain. Microbubbles (BR1, research grade equivalent to SonoVue®) and liposomes were administered intravenously via a catheter inserted into the tail vein at a 1:4 dilution with a total bubble dose 2 × 10^7^ per injection. Mice were then treated using the following sonication parameters: peak-negative pressure: 320 ± 30 kPa, centre frequency: 0.5 MHz, pulse length = 500 cycles = 1 ms, pulse repetition frequency = 5 Hz, total number of pulses = 600.

MRI was performed at 2 and 4 h post sonication to evaluate contrast enhancement due to liposome delivery across the BBB. We acquired T_1_ weighted RARE (TR/TE = 4.26/600 ms, averages = 4, FOV = 20 × 20 mm, matrix size 128 × 128, slice thickness = 1 mm) and T_2_ weighted TURBORARE (TR/TE = 2500/30 ms, averages = 4, FOV = 20 × 20 mm, matrix size 256 × 256, slice thickness = 1 mm) scans, to evaluate the efficacy and safety of FUS treatments. Animals were sacrificed after the 4-h scan through transcardial perfusion and their brains were excised for fluorescence imaging and histology. Briefly, animals were intraperitoneally injected with a terminal dose of pentobarbital, their limbs splayed in the supine position. We performed an incision to cut through skin, muscle wall and ribs to reveal the heart. The vena cava was then identified and cut before slowly administering 10 mL saline followed by 10 mL 4 % paraformaldehyde into the left ventricle.

### 2.7 Signal processing/cavitation analysis

Acoustic cavitation emissions were processed offline using Matlab® (Mathworks, MA, USA) similarly to previous studies [34,36]. Briefly, the energy emitted from exposed microbubbles during a single therapeutic pulse was estimated from the time domain signal through:

(1) $$E∼\int_{0}^{T}V^{2}dt\approx \sum_{t=0}^{T}V^{2}\Delta t$$

where V was the voltage at each time point in volts and the sampling period Δt was 1.25 × 10^−8^ s. The electrical energy detected was assumed to be proportional to the acoustic energy emitted by microbubbles. Control sonications before microbubble administration were used to estimate baseline acoustic signal, whose energy was subtracted at each time point.

Frequency analysis was used to identify the dominant cavitation mode for each treatment. Three spectral areas from the Fourier Transform (harmonic region, ultraharmonic region, and broadband regions) were filtered and identified:

(2) $$harmonic\;regions,f_{h,n}=nf_{c}$$

(3) $$ultraharmonic\;regions,f_{u,n}=\left(n+0.5\right)f_{c}$$

$$broadband\;regions,f_{b}=f_{h,n}+10\;kHz<\;f_{b}<\;f_{u,n}-10\;kHz\;and$$

(4) $$f_{u,n}+10\;kHz<\;f_{b}<\;f_{h,n+1}-10\;kHz$$

where $f_{c}$ was the center frequency of the FUS transducer and n is the harmonic number. The fundamental and second harmonic were filtered out using a high pass filter due to strong reflections at these frequencies in control pulses. Cavitation doses were calculated based on the root-mean-square voltage detected in the retrospective areas [11]. The stable harmonic (SCD_h_), ultraharmonic (SCD_u_) and inertial (ICD) cavitation doses were defined as:

(5) $${CD}_{i}=\sqrt{{\left\langle {\left|FFT\right|}_{f_{i}}^{2}\right\rangle }_{n}}$$

where i changes for harmonic (n = 3 to 10), ultraharmonic and broadband (inertial) regions.

### 2.8 Image processing for MRI quantification

MRI images were processed in Matlab©. Quantification was performed on both coronal and axial slices. A region of interest (ROI) was defined in the contralateral hemisphere to determine baseline signal intensity. The threshold intensity for defining BBB opening was set as two standard deviations (95 % confidence) above this baseline. Every axial and coronal slice was loaded sequentially, and an ROI was manually drawn around the entire ipsilateral hemisphere. All pixels with intensities higher than the threshold were counted to derive the surface area of liposome delivery in each slice, and were grouped into contour plots to facilitate visualisation. The volume of liposome delivery was calculated for axial and coronal slices separately by multiplying by the slice thickness (1 mm) and averaged to estimate the total volume of liposome-induced contrast enhancement. Finally, the contrast enhancement (in %) was calculated by dividing the mean intensity within the areas of liposome delivery areas with the mean intensity of the control ROI. Two animals were excluded from linear correlations between MRI volume and PCD metrics due to the presence of significant T2 image abnormalities.

### 2.9 Histology

Mice were sacrificed immediately after the final MRI time point. A transcardial perfusion was performed using 10 mL of PBS followed by 10 mL of 4 % paraformaldehyde (PFA). The brains were removed and fixed in 4 % PFA for 4 h before being transferred into a 30 % sucrose solution for cryopreservation for at least 24 h. Brains were embedded in OCT and coronally sectioned into 10 μm slices from an anterior to posterior direction. Brain slices were mounted on glass slides and stained with hematoxylin and eosin (H&E) to assess microscopic damage and blood vessel integrity. Images were acquired using an AxioScan Z1 (ZEISS, Germany).

### 2.10 Statistics

Measurements are presented as mean ± standard deviation throughout the manuscript. A two-tailed *t*-test was performed to compare liposome delivery volume and contrast enhancement. Linear regression was performed to correlate liposome delivery metrics with cavitation doses. Statistical significance was defined for p < 0.05.

## 3 Results

### 3.1 Liposome MRI phantom optimization

**Fig. 2:**
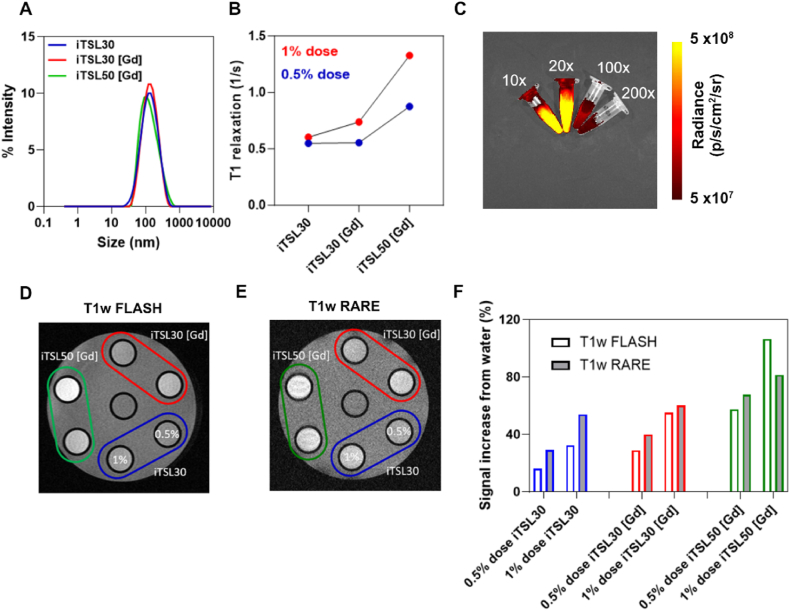
iTSL MRI/NIRF optimization A. Size distribution of iTSL. B. T1 relaxation rates of iTSL's at 1 % and 0.5 % of the administered dose respectively. C. Fluorescence signal at 10-, 20-, 100- and 200-fold dilution of injected dose. D-E. Example T_1_-weighted MRI images of the three iTSL formulations at 1 and 0.5 % of injected dose concentration. F. Contrast enhancement of iTSLs compared to water, using FLASH and RARE T_1_-weigthed MRI sequences.

In this study, we prepared imageable liposomes that have both NIRF and MRI contrast lipid conjugates incorporated into the lipid bilayer. The lipid formulation was DPPC: DSPC:MSPC: DSPE-PEG*2000*OMe: [Gd]DOTA.DSA: CW800.DSA at 53.9:5:5:6:3:0.1 mol%. The contrast agent lipids [Gd]DOTA.DSA (MRI contrast) and CW800.DSA (NIRF contrast) were synthesized in house following previously reported procedures [20,31]. Fig. 2A shows that incorporation of Gadovist™ into the aqueous core has minimal effect on the hydrodynamic size of the formulations with sizes of 115 ± 0.9 nm, 125 ± 3.1 nm, and 114.6 ± 3.2 nm for iTSL, iTSL30[Gd], and iTSL50[Gd], respectively. T_1_ weighted FLASH (Fig. 2D) and RARE (Fig. 2E) MRI phantom images were generated for the three iTSL formulations at concentrations equivalent to 1 % and 0.5 % of the injected dose. As shown in Fig. 2F, the percentage increase in signal of iTSL's compared to water is higher using a RARE imaging sequence and increases with both encapsulation of Gadovist (iTSL30[Gd]) and increasing lipid concentration from 30 mg/mL to 50 mg/mL (iTSL50[Gd]). Fig. 2B shows the highest relaxation was measured for iTSL50 [Gd]. The fluorescence of iTSL can be detected at concentrations as low as 0.05 % of the administered dose (Fig. 2C). The relaxivity of each liposome formulation was also calculated from T_1_ mapping and gadolinium concentration which was determined using ICP-OES and is summarized in Table 1. Based on these phantom measurements, it was decided to use the iTSL50 [Gd] and RARE imaging for *in vivo* measurement of liposome delivery to the brain as this combination provided the highest contrast.

**Table 1:** Nanoparticle characteristics: Representative colloidal parameters for empty (iTSL) and Gadovist™ loaded liposomes iTSL-Gd30 and iTSL-Gd50. Sizing data was measured using dynamic light scattering (DLS); Gadolinium concentration and % Gadovist™ encapsulation was determined using ICP-OES with D/L (Drug/Lipid) ratios calculated for encapsulated Gadovist™ vs total lipid concentration; relaxivity was calculated from MRI phantom T_1_ maps. All values are mean average ±1 SD; n = 3, for purified liposome formulations; PDI, polydispersity index; D/L, drug/lipid ratio in the final liposome.

Liposome	Size (nm)	PDI	[Gd] (mg/ml)	Longitudinal relaxivity r1 (mM^−1^s^−1^)	Gadovist™ encapsulation (%)
iTSL	115 ± 1	0.25 ± 0.01	1.13 ± 0.19	3.32 ± 0.05^18^	–
iTSL-Gd30	117 ± 3	0.18 ± 0.01	3.00 ± 0.13	2.61 ± 0.36	1.16 ± 0.08 (D/L = 0.23 ± 0.01)
iTSL-Gd50	111 ± 4	0.18 ± 0.01	5.21 ± 0.18	2.71 ± 0.61	2.07 ± 0.12 (D/L = 0.25 ± 0.01)

### 3.2 MRI confirmation and quantification of liposome delivery

**Fig. 3:**
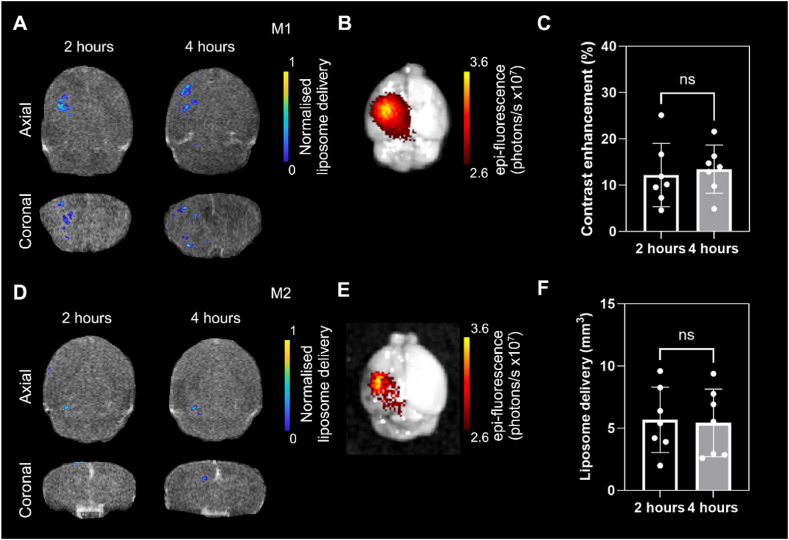
Multimodal confirmation of iTSL delivery to the brain. A and D, Hyperintense regions in T_1_w MRI indicate liposome delivery at 2 and 4 h post sonication. B and E, *Ex-vivo* fluorescence images confirmed location of liposome delivery after FUS. C. Brain volume with contrast enhancement due to delivered liposomes. F. MRI contrast enhancement resulting from liposomes after FUS.

To investigate if FUS-induced BBB opening facilitates liposome uptake into the brain, the left hippocampus was treated using a 0.5 MHz transducer, 320 ± 30 kPa peak-negative pressure, while intravenously injecting BR1 microbubbles (research grade equivalent to SonoVue®, dose = 2 mL of BR1 solution/kg) combined with liposomes (50 mg/mL at a dose of 8 mL/kg or 400 mg/kg, n = 7). To determine the spatiotemporal distribution of liposomal uptake, MRI was carried out at 2 and 4 h post-ultrasound treatment. Hyperintense regions were observed in coronal and axial MRI images (Fig. 3A–B, D-E) confirming liposome delivery at both 2 and 4 h. Delivery at the targeted location was confirmed *ex vivo* using NIRF imaging (Fig. 3C, F and SF2). In general, when BBB opening spread throughout a wider area (Fig. 3A-B), liposome distribution appears to become more diffuse, potentially beginning to clear from the focus at 4 h. However, for BBB opening over a smaller volume (Fig. 3D-E), liposomes appear to accumulate between 2 and 4 h. As expected, no MRI contrast or fluorescence was detected in the control right hippocampi of all brains. The mean liposome delivery volume and liposome-induced contrast enhancement was 5.7 ± 2.6 mm^3^ and 12.2 % at 2 h, 5.4 ± 2.7 mm^3^ and 13.4 % at 4 h (n = 7). No statistically significant difference was observed between the two time points (P = 0.86 and P = 0.70 respectively, two-tailed *t*-test). While there appears to be a large range in the contrast-enhanced area and contrast enhancement, no significant difference was found between 2 and 4 h post FUS treatment. T_2_ changes were observed in all mice (Suppl. Fig. 1). The two mice with the lowest cavitation dose, and treated at pressures of 300 kPa, had small hyperintense regions which can be associated with mild cerebral edema [37]. Additional changes were observed in the other 5 mice (four at 320 kPa, one at 350 kPa). In all cases, the degree of change observed in T_2_ images reduced between 2 and 4 h, indicating the brain is undergoing recovery immediately after FUS treatment. Interestingly, T_1_ and T_2_ enhancements occur at different locations, with liposomes typically accumulating around the perimeter of the T_*2*_ contrast enhanced spot.

### 3.3 FUS liposome delivery correlates with microbubble acoustic emissions

**Fig. 4:**
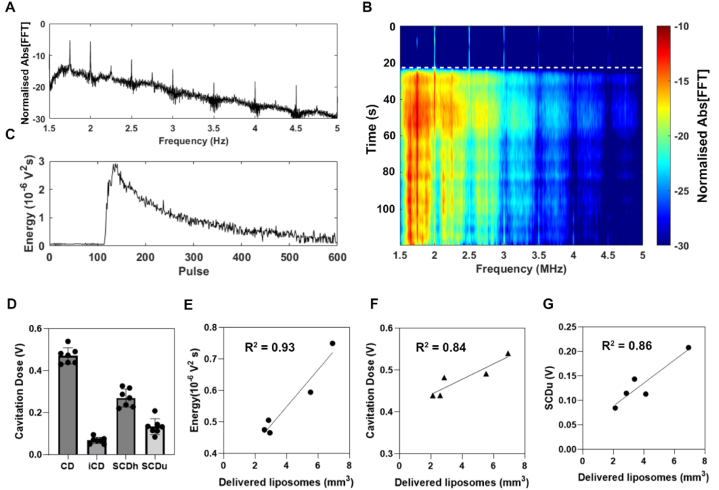
Total energy and cavitation dose correlate with liposome-induced contrast enhancement volume. A. Normalized amplitude of Fast Fourier Transform (FFT) performed over acoustic cavitation emissions. B Spectrogram of FUS treatment (white dashed line indicates when microbubbles enter the focal volume). C. Total energy per therapeutic pulse. D. inertial (ICD), stable harmonic (SCDh), stable ultraharmonic (SCDu) and total cavitation dose (CD) per animal E-G. linear correlation between delivered liposome volume and total acoustic energy, total cavitation dose and ultraharmonic cavitation dose respectively.

Passive cavitation detection was performed on all mice for real-time monitoring of BBB opening and to investigate potential correlations with MRI-derived metrics. Spectral analysis was performed through Fast Fourier Transform (FFT) of time-domain cavitation signals (Fig. 4 A-B). We observed persistent harmonic emissions throughout the treatment duration, indicative of stable cavitation, as well as moderate broadband emissions, indicative of inertial cavitation (Fig. 4B). The detected energy increased upon entry of microbubbles into the focal volume and then exponentially decreased due to microbubble clearance from circulation (Fig. 4C). The mean energy calculated from acoustic emissions was 5.1 ± 0.14 × 10^−5^ V^2^s and was broadly consistent across mice (n = 7, Suppl. Fig. 2). The mean cavitation dose was 0.47 ± 0.04 V, 86 % of which from stable cavitation and 14 % inertial cavitation (Fig. 4 D). Total acoustic energy (Fig. 4 E), total cavitation dose (Fig. 4 F), and stable ultraharmonic cavitation dose (Fig. 4 G) correlated linearly with delivered liposome volume observed from MRI with R^2^ = 0.93, 0.84 and 0.86, respectively (n = 5). The stable harmonic cavitation dose (SCDh), stable harmonic cavitation dose (SCDh + SCDu), and inertial cavitation doses did not correlate significantly with delivered liposome volume (R^2^ = 0.18, 0.14 and 0.52 respectively, Suppl. Fig. 2).

### 3.4 Histology

**Fig. 5:**
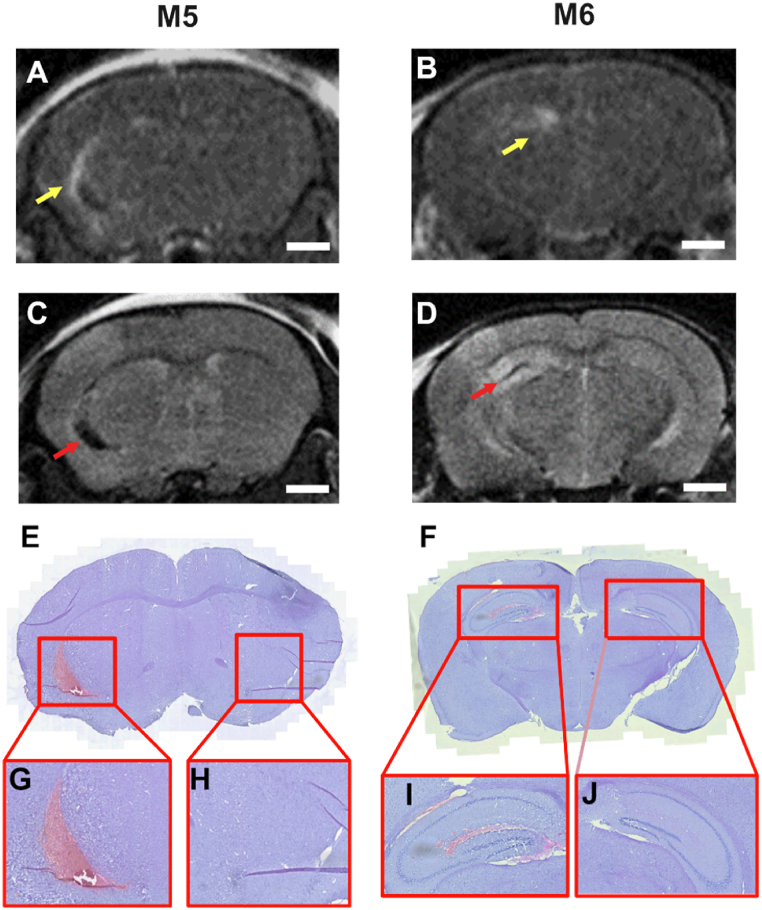
A-B. T_1_ weighted MRI showing hyperintense regions as a result of liposome delivery (yellow arrows). C-D. Corresponding T_2_ weighted MRI showing hyper and hypointense regions (red arrows) at the sonication region. E – F. Corresponding histology brain slices. G, I. 20× magnification showing hemorrhage and extravasation of red blood cells respectively. H, J. 20× magnification showing non-sonicated hemisphere is unaffected. (For interpretation of the references to color in this figure legend, the reader is referred to the Web version of this article.)

Histological staining of coronal brain slices revealed that hypointense regions observed in T_2_ weighted MRI was associated either with micro-hemorrhage (Fig. 5C, E and 5G) or liposome extravasation (Fig. 5D, F and 5I) depending on the location and the size of hypointensity. No structural differences were observed in hyperintense regions that could be correlated with the treatment. The contralateral non sonicated hemisphere was also unaffected (Fig. 5H and J). When hypointense areas were observed in T_2_ weighted images, contrast enhancement from delivered liposomes in the T_1_ weighted images appear around the perimeter of these locations (Fig. 5A and B).

## 4 Discussion

Liposomes have previously been reported to induce an MRI contrast enhancement and a therapeutic effect in subcutaneous murine tumor models [20,31,32]. In this study, we demonstrated that these liposomes can be successfully delivered to the brain of wild-type mice through FUS-induced BBB opening. Liposomes 110 nm in diameter with gadolinium in the lipid and in the aqueous core were detected in treated areas using MRI, allowing confirmation of their delivery in a defined region of the brain. All treated mice had detectable hyperintense regions at the location of FUS-induced BBBO (Fig. 3). These areas were visible at 2- and 4-h post-treatment. NIRF imaging at 4 h post treatment confirmed the presence of liposomes in the same brain location *ex vivo* in perfused brains. NIRF imaging of the liposomes in the brain is challenging in a live animal due to the presence of the skull that obstructs NIRF signal. In addition, the brains had to be perfused to remove signal from circulating liposomes in the brain blood vessels that interfere with the signal coming from the sonicated area. The dual ability to detect delivery as well as visualize their distribution within the brain can be used to understand the mechanism of BBBO and provide treatment guidance to clinicians.

Previous studies have shown that delivery efficiency is greater using liposomes with smaller diameters [7,24,38] and they remain at the site of treatment 24 h post FUS. While sub-100 nm liposomes may have higher penetration through the disrupted BBB, and potentially provide higher MRI contrast, they are limited on their amount of hydrophilic drug cargo, due to the smaller volume of the aqueous core. In this study, liposomes 110 nm in size were chosen as they have already been shown to exhibit promising drug delivery efficacy and therapeutic effect in pre-clinical cancer models [20,31]. These liposomes were designed to be imageable (Gd.DOTA.DSA lipids) and to carry two drugs; carboplatin in the aqueous core and SN38 in the membrane. These iTSLs can deliver drugs after short application of mild hyperthermia via a suggested two-fold mechanism. First the liposomes release carboplatin rapidly after the lipid membrane pore formation due to hyperthermia, then the remaining lipid bilayer with the SN38 forms small micelles that are taken up by cells rapidly. Their thermosensitive nature allows for more targeted drug release that occurs rapidly inside the tumor. However, choosing the timing of mild hyperthermia after administration is crucial for delivering the maximum therapeutic payload. The same FUS transducer used for BBB opening can also be used to generate mild hyperthermia (e.g. few degrees to reach 41 °C for 5–10 min) by adapting ultrasonic parameters such as acoustic pressure, duty cycle and pulse length. As such, the treatment could be completed within few minutes and in one session. An additional advantage is that mild hyperthermia alone has been shown to enhance sensitivity to treatments and drug response [39]. However, microbubbles must be cleared from the vasculature beforehand to avoid damage caused when a high mechanical index is applied. Microbubbles have been shown to clear within minutes [40], whereas good accumulation of liposomes was observed at 2 and 4 h, similar to Aryal et al. [24], providing an adequate time window between FUS-induced delivery and triggered drug release, which is essential for enhanced efficacy without compromising the safety of treatments. MR-based or ultrasound-based thermometry could be introduced in investigations to control the procedure, by estimating the localized temperature increase within treated areas for real-time safety monitoring.

Contrast enhanced T_1_ weighted MRI is frequently used to assess BBB opening with gadolinium chelates being the contrast agent of choice [37]. While Gd-enhanced MRI provides an indication of the magnitude and distribution of BBB opening, it is not a direct confirmation of drug delivery. Gadolinium chelated small molecular contrast agents have pharmacokinetics that are different to drugs or their carriers. The use of gadolinium labelled liposomes overcomes this, however the pros and cons and potential of these theranostic liposomes needs further assessment. The gadolinium concentration in this study is approximately 30 times less than clinically approved agents such as Gadovist, even at the 50 mg/mL formulation. To this end, we showed that using a RARE spin-echo sequence can enhance the liposome contrast at concentrations expected to reach the brain after BBB opening (0.5–1% injected dose), compared to the commonly used T_1_ weighted FLASH sequence. Interestingly, the FLASH sequence provided higher contrast at the highest concentration (Fig. 2F), indicating that this sequence is preferable when administering gadolinium contrast agents and imaging using high strength magnets. It is still uncertain whether the gadolinium and fluorescent lipids detected in the brain were still encapsulated in the liposomes and how far from the blood vessels they penetrated into the parenchyma. Further work is needed to evaluate the dynamics of liposome delivery into brain tissue. For example, DESI-MS could be used to investigate lipid distribution and compared to MRI slices [41].

In all cases, the degree of contrast change observed in T_2_ weighted images reduced between 2 and 4 h, indicating the brain is undergoing recovery immediately after FUS treatment, even at a relatively high mechanical index. Further optimization studies are needed to define a safe range of ultrasound parameters and microbubble doses, so that the risk-benefit ratio is carefully balanced when attempting to deliver large drugs and drug vehicles, such as viral vectors or liposomes (diameter >100 nm). Future work on carrier delivery, drug distribution and BBB recovery dynamics of acute brain imaging changes should evaluate the degree of acceptable and reversible level of structural changes the brain can undergo (Fig. 5), especially within diseased tissue (e.g., GBM or DMG). In animals, histology is often adopted to assess safety, typically around 24 h post-FUS [24,42]. However, Sun et al. observed T2 enhancement up to 5 days post-FUS [37]. Small areas of hypo-intensities in T_2_∗ weighted imaging have also been correlated to vascular damage in primates, with 50 % occurrence at pressures around 358 kPa. However, no behavioral changes were observed in animals where hypointense regions were present, even up to 6 months post initial treatment [43].

It is likely that the delivered liposome MRI volumes calculated are an underestimate of the true amount due the relatively low contrast of the liposomes compared to free gadolinium contrast agents used in other preclinical studies. To increase sensitivity, further MRI optimization could also be performed. A surface coil could be used to increase signal-to-noise and contrast-to-noise ratios, however the signal drop-off with distance from the coil may affect quantification of delivery. Transitioning from a 9.4 T to a 3 T MRI scanner will also help due to the increased relaxivity at the lower clinically used field strengths [44] and enhance scalability and clinical relevance of iTSLs.

Passive cavitation detection was performed during *in vivo* sonications for real-time monitoring of safety and successful BBB opening (Fig. 4). The total acoustic energy, total cavitation dose and stable ultraharmonic cavitation dose correlated positively with MRI signal increase, whereas inertial cavitation dose and total stable cavitation dose did not, in contrast to the results reported in Sun et al. [37]. O'Reilly et al. [15] used ultraharmonics emissions in a semi-closed loop control system for real-time monitoring and control of BBB opening, however consistent detection of ultraharmonic activity depends on the type of PCD system used [16]. Passive acoustic mapping may be preferred to one dimensional PCD systems as it provides more spatial information [45]. Nonetheless the correlation between cavitation response and MRI signal indicates cavitation activities can be used to monitor liposome delivery. It appears that it is important to correlate liposomal delivery with passive acoustic mapping measurements.

Integrated MRI-FUS systems are often used that deliver an array of multiple sonications to deliver drugs to a wider area [24,46,47] which lengthens the total procedure time [48]. Here, for this study in mice, we used a single element transducer with a focal width of 2.3 mm, enabling the delivery of liposomes to the brain over a typical mouse brain tumor volume with a single sonication. The focal volume can be further broadened to more clinically relevant focal volumes either using transducers with wider apertures [11] or acoustic holograms [49], to enhance the clinical relevance of this setup. The reduced time of this procedure and less MRI time has the potential to make FUS-BBB opening more accessible to patients. Future work will include the delivery of drug-loaded imageable liposomes in murine models of adult and pediatric brain tumors. In addition, MR-based contrast enhancement and passive cavitation measurements will be correlated with the delivered chemotherapeutic doses, to establish methods for accurately evaluating the quantity of drugs reaching the brain tumor following FUS treatment.

To summarize, this study demonstrates reliable quantification of the delivery of 110 nm liposomes to the brain after FUS-induced BBB opening using MRI, providing a detailed evaluation of the spatial-temporal distribution of liposomes in the brain up to 4 h post treatment. This, along with a correlation of MRI signal to acoustic emissions during treatment could provide doctors and patients immediate feedback on treatment success. This study also provides a framework on which to build for future studies, particularly on delivery of drug loaded liposomes in orthotopic brain tumor models. The thermosensitive nature of these liposomes will also be utilized for more targeted drug release. This combined image-guided drug delivery and release technology has the potential to overcome the major obstacles in brain tumor treatments.

## CRediT authorship contribution statement

**Chris Payne:** Conceptualization, Data curation, Formal analysis, Investigation, Methodology, Software, Validation, Writing – original draft, Writing – review & editing. **Paul Cressey:** Conceptualization, Investigation, Methodology, Writing – original draft. **Anisia Talianu:** Investigation, Visualization. **Elwira Szychot:** Conceptualization, Funding acquisition, Writing – review & editing. **Darren Hargrave:** Funding acquisition, Writing – review & editing. **Maya Thanou:** Conceptualization, Funding acquisition, Project administration, Resources, Supervision, Writing – review & editing. **Antonios N. Pouliopoulos:** Conceptualization, Funding acquisition, Project administration, Resources, Software, Supervision, Writing – original draft, Writing – review & editing.

## Declaration of competing interest

Dr. Maya Thanou is a co-founder of the King’s College London spinout Apeikon Therapeutics that holds patents related to the technology discussed in this work. Apeikon Therapeutics is an industrial partner, who collaborated with King’s College London forming agreements for managing background IP and licensing novel technologies used in the published research. The remaining authors declare no conflicts of interest.

## Figures and Tables

**Suppl. Fig. 3:**
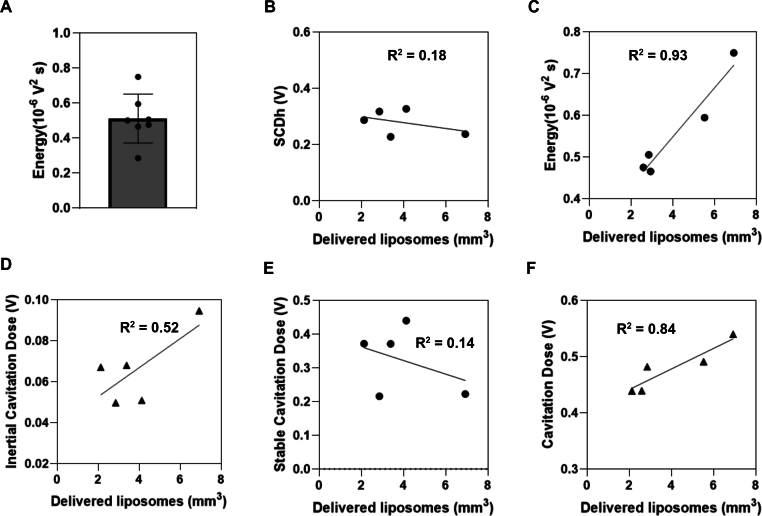
A) Total acoustic energy, B) Stable harmonic cavitation dose vs MRI signal, C) total acoustic energy vs MRI signal, D) inertial cavitation dose vs MRI signal, E) stable cavitation dose (harmonic and ultraharmonic) vs MRI signal, F) total cavitation dose vs MRI signal.

## Appendix A Supplementary data

Fig. S1 T_1_ weighted and T_2_ weighted images (coronal and axial slices) of all animals. Fig. S1

Fig. S2 *Ex vivo* whole brain NIRF images. Fig. S2
