# Supplementary figures and images for: Bi-modal confirmation of liposome delivery to the brain after focused ultrasound-induced blood-brain barrier opening
Source: Heliyon. 2024 Nov 6;10(22):e39972. doi: 10.1016/j.heliyon.2024.e39972 (PMC11609457; doi:10.1016/j.heliyon.2024.e39972)

Mouse 1

T1 T2


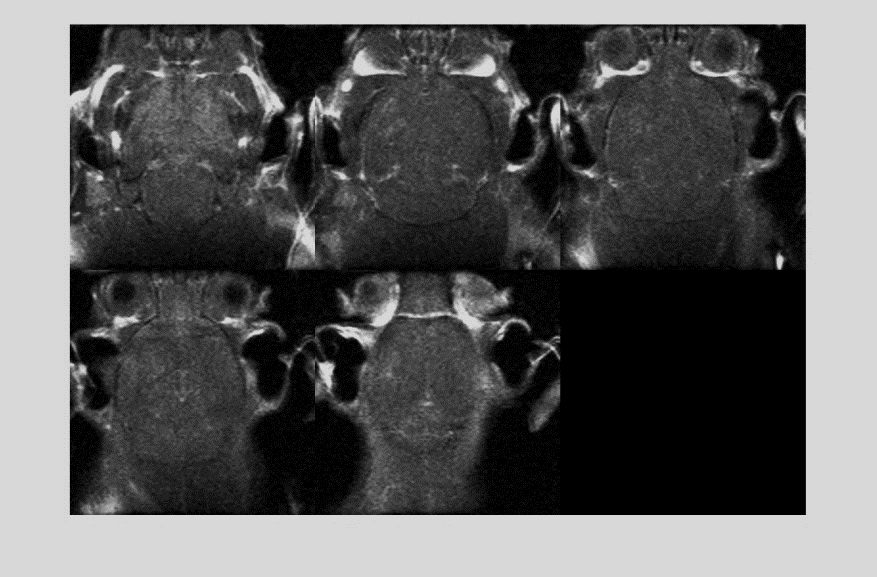

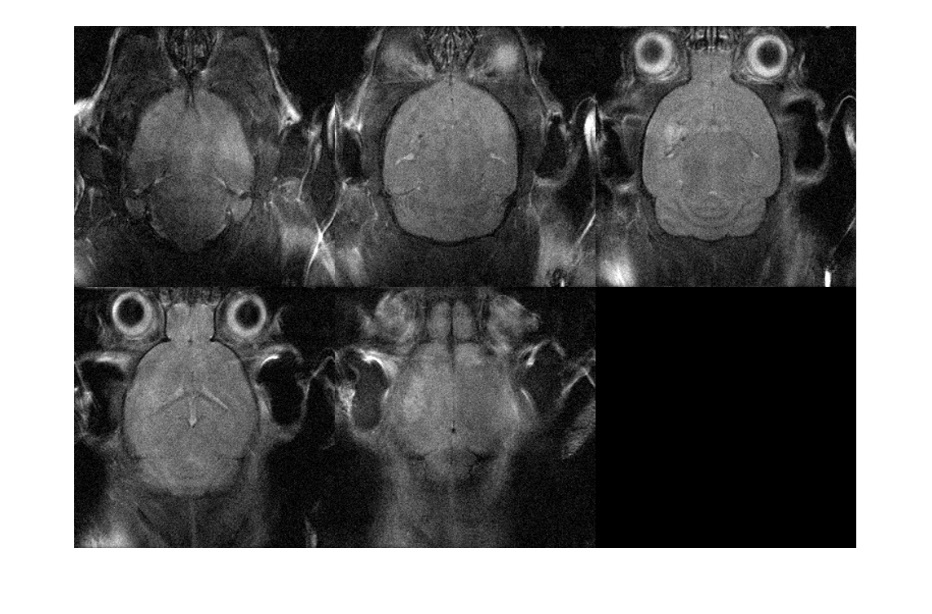


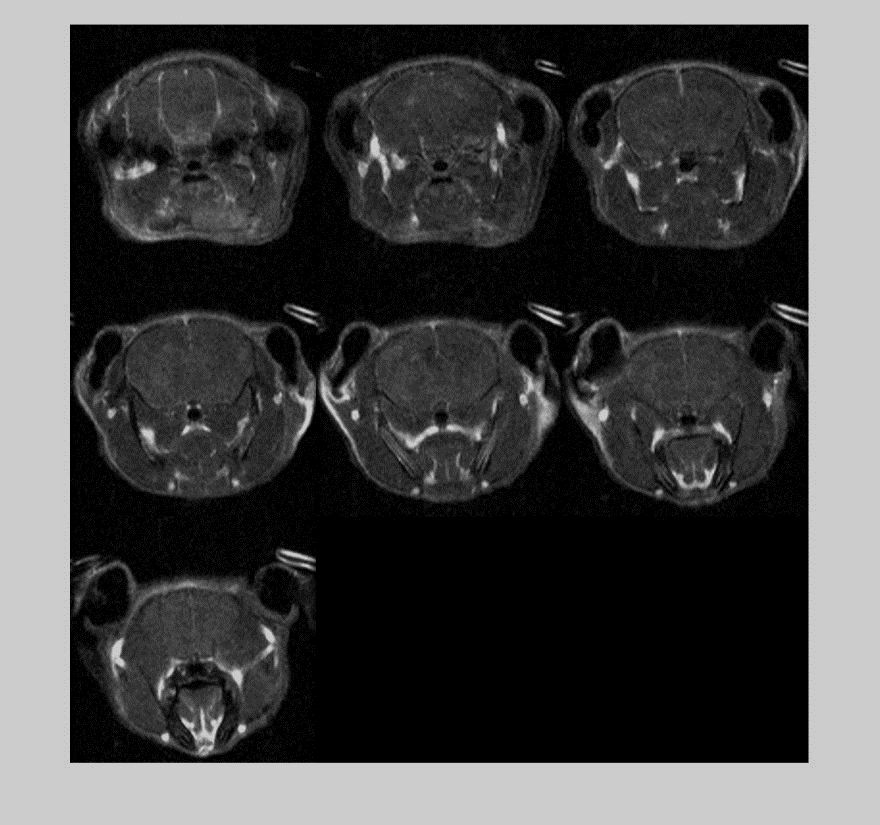

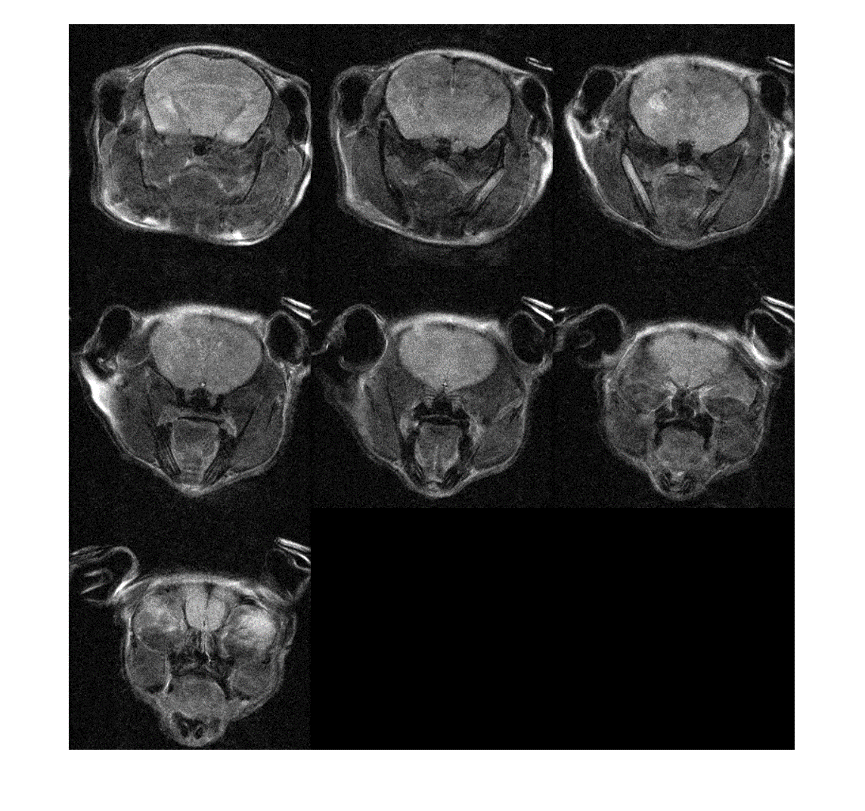


Mouse 2

T1 T2


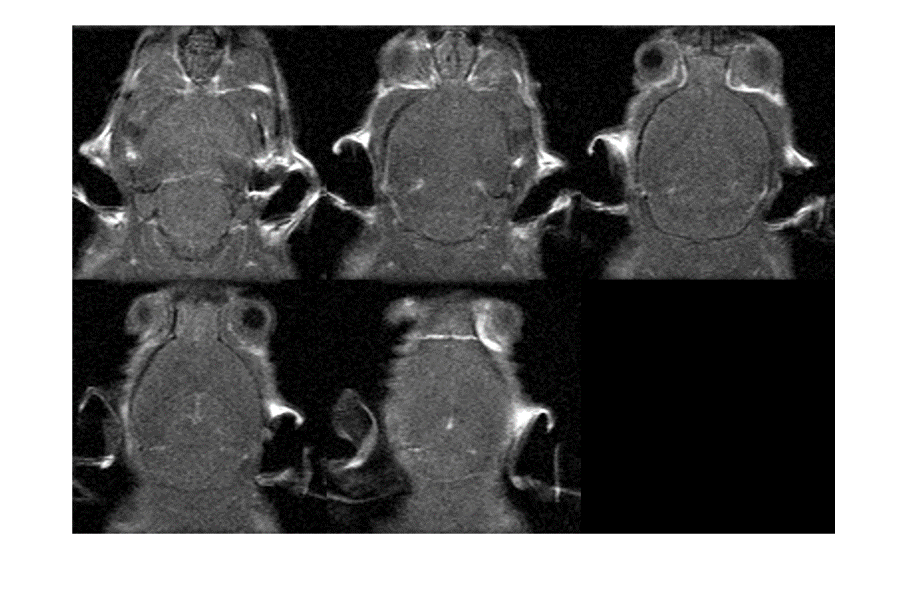

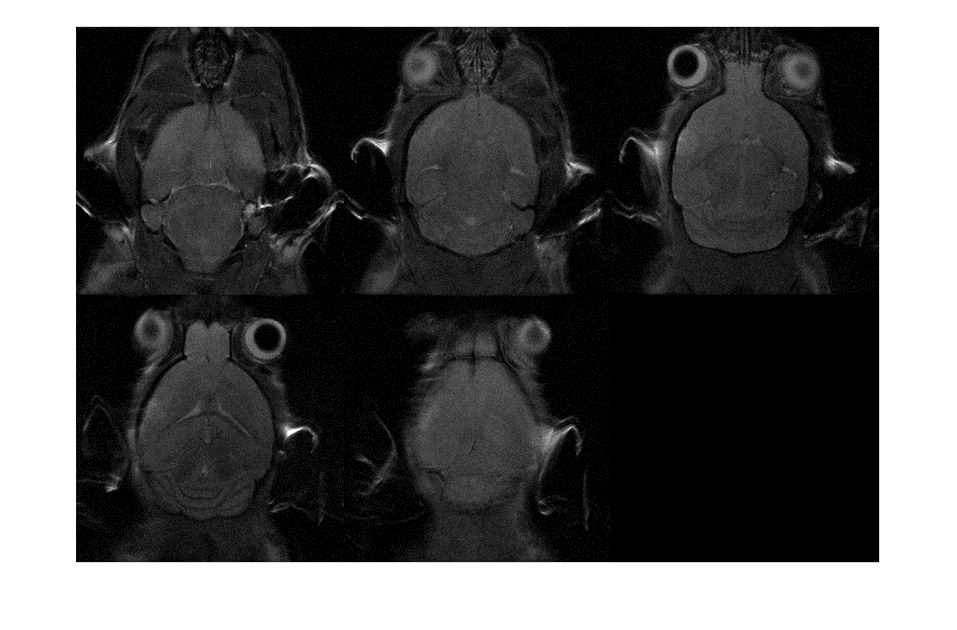


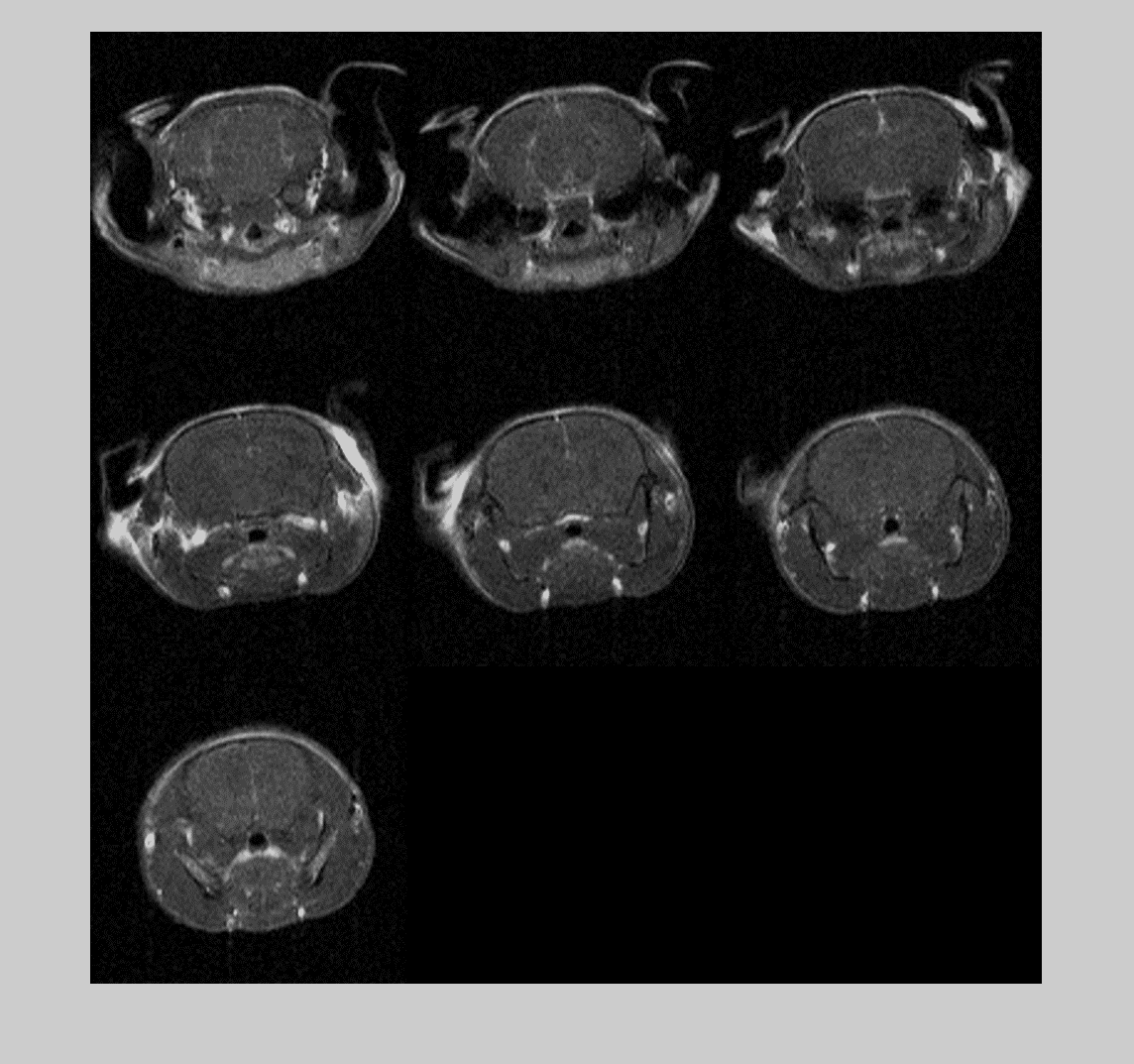

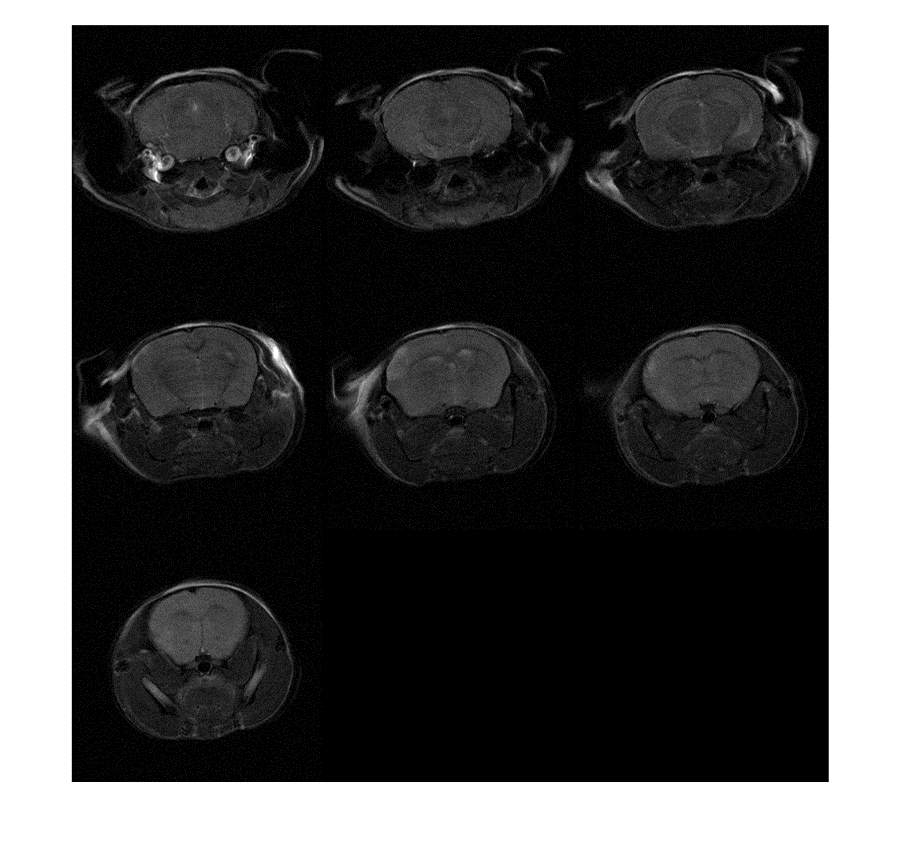


Mouse 3

T1 T2


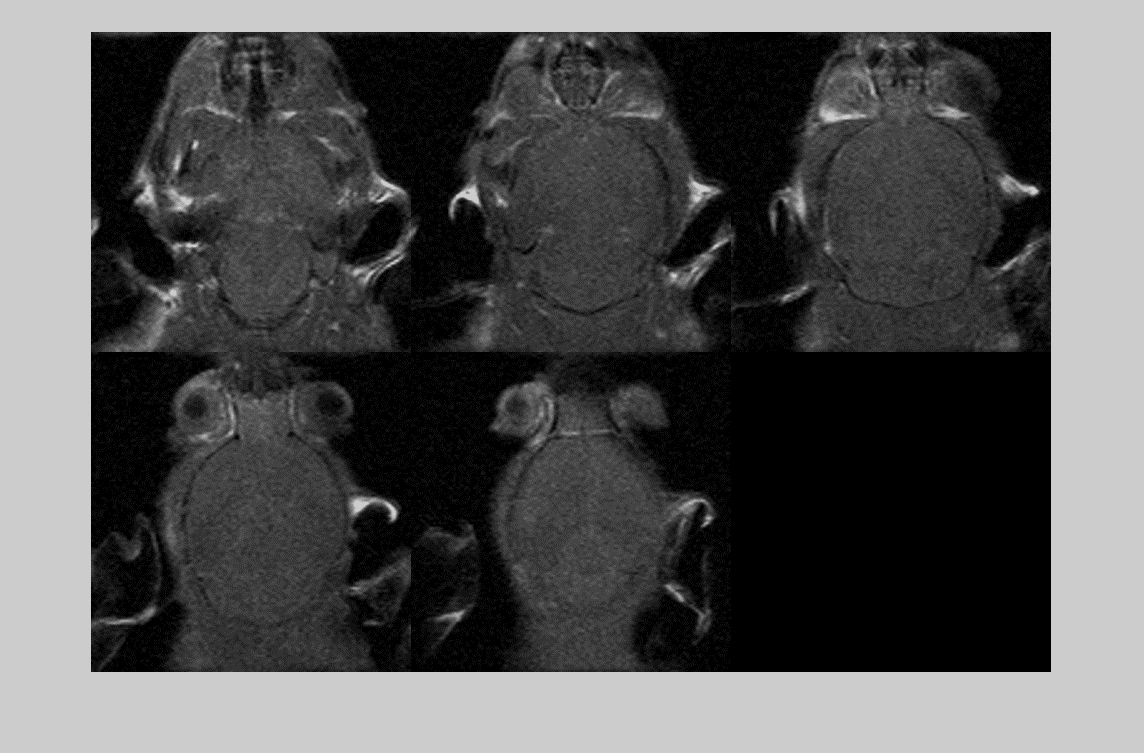

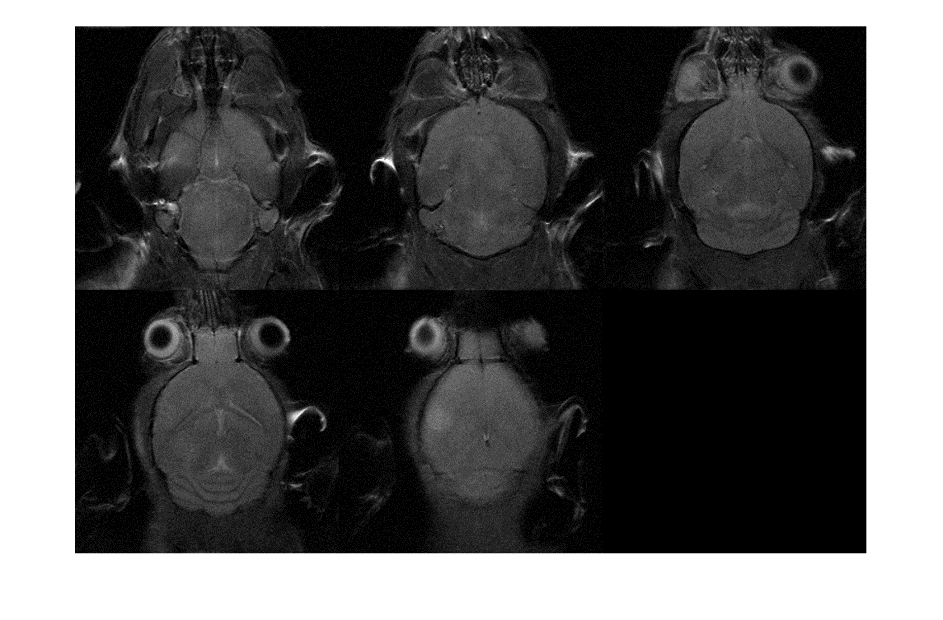


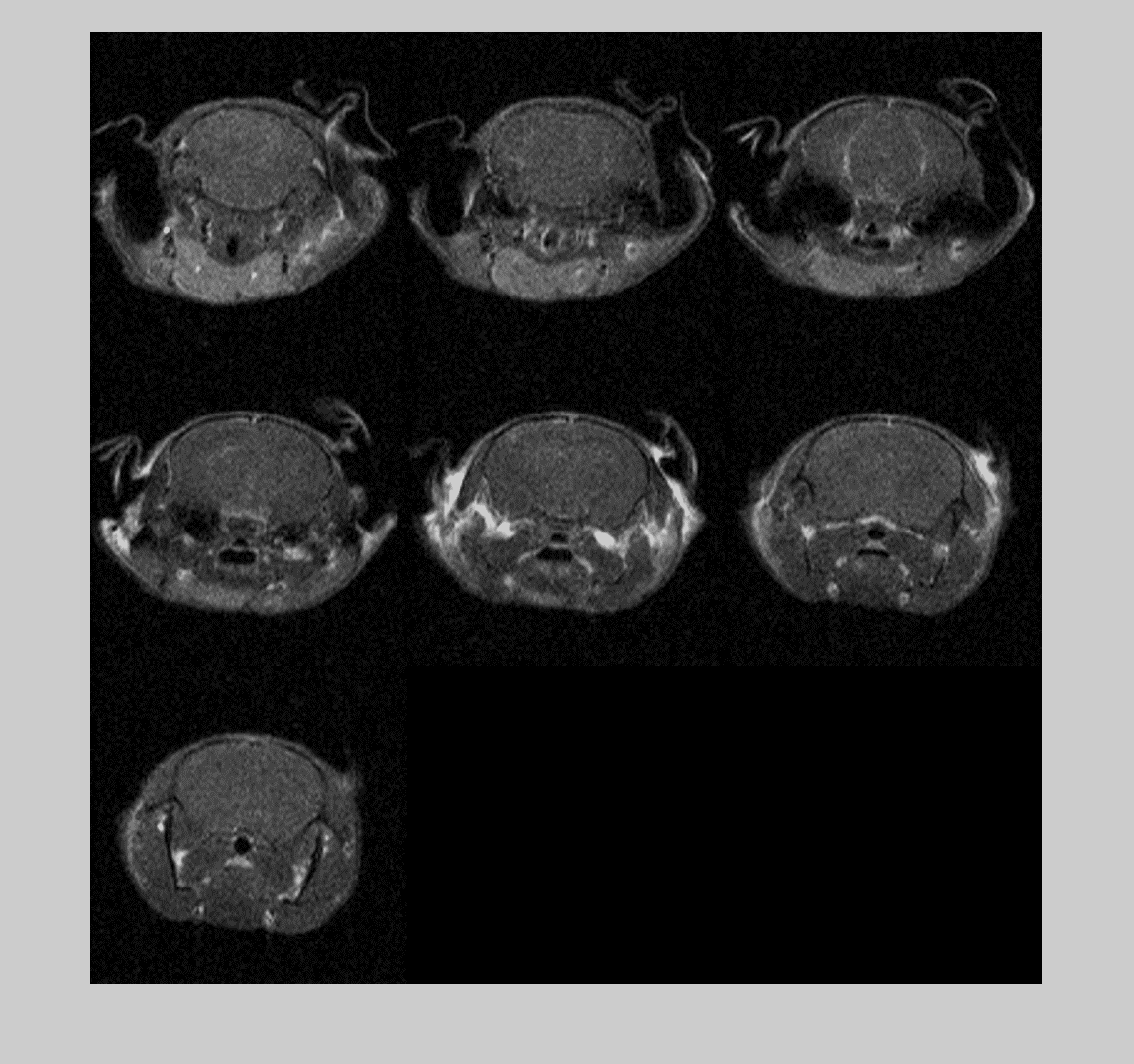

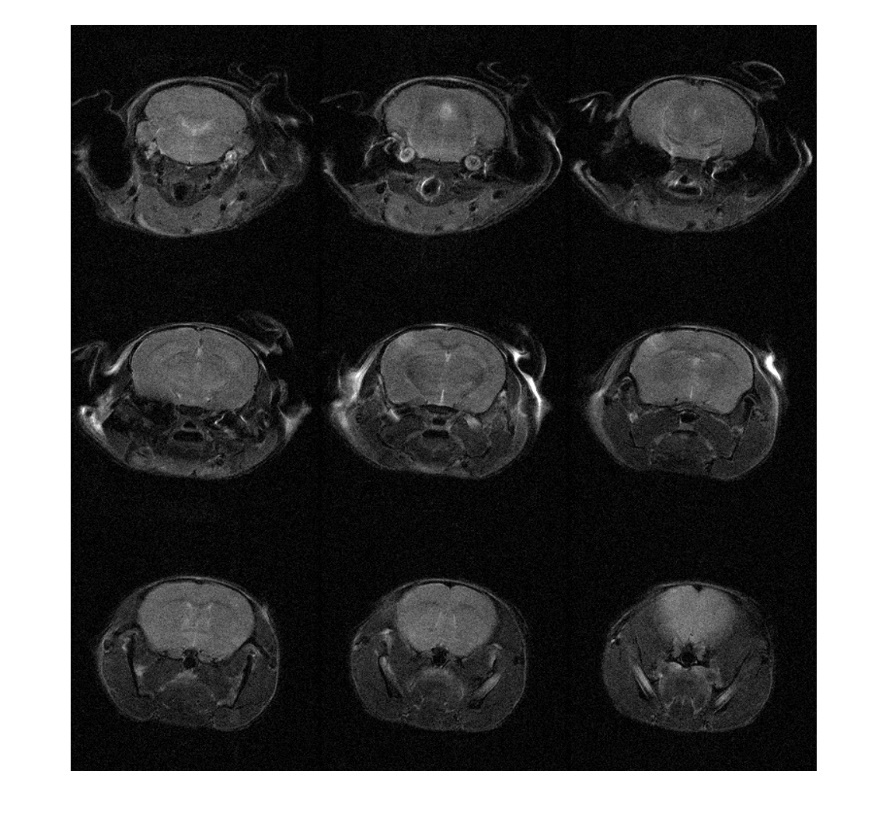


Mouse 4

T1 T2


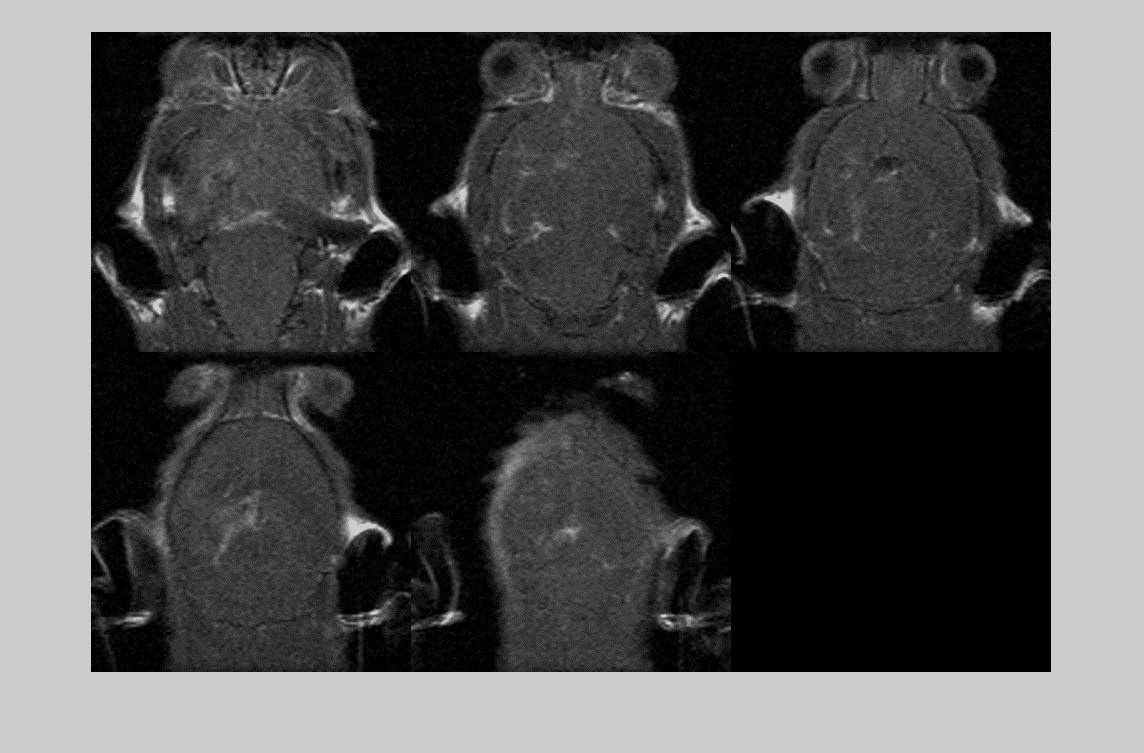

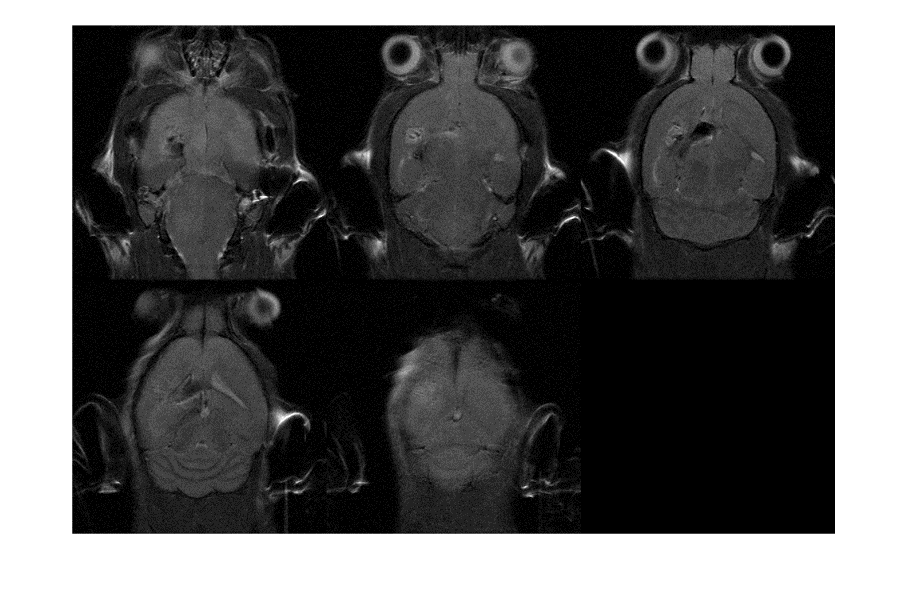


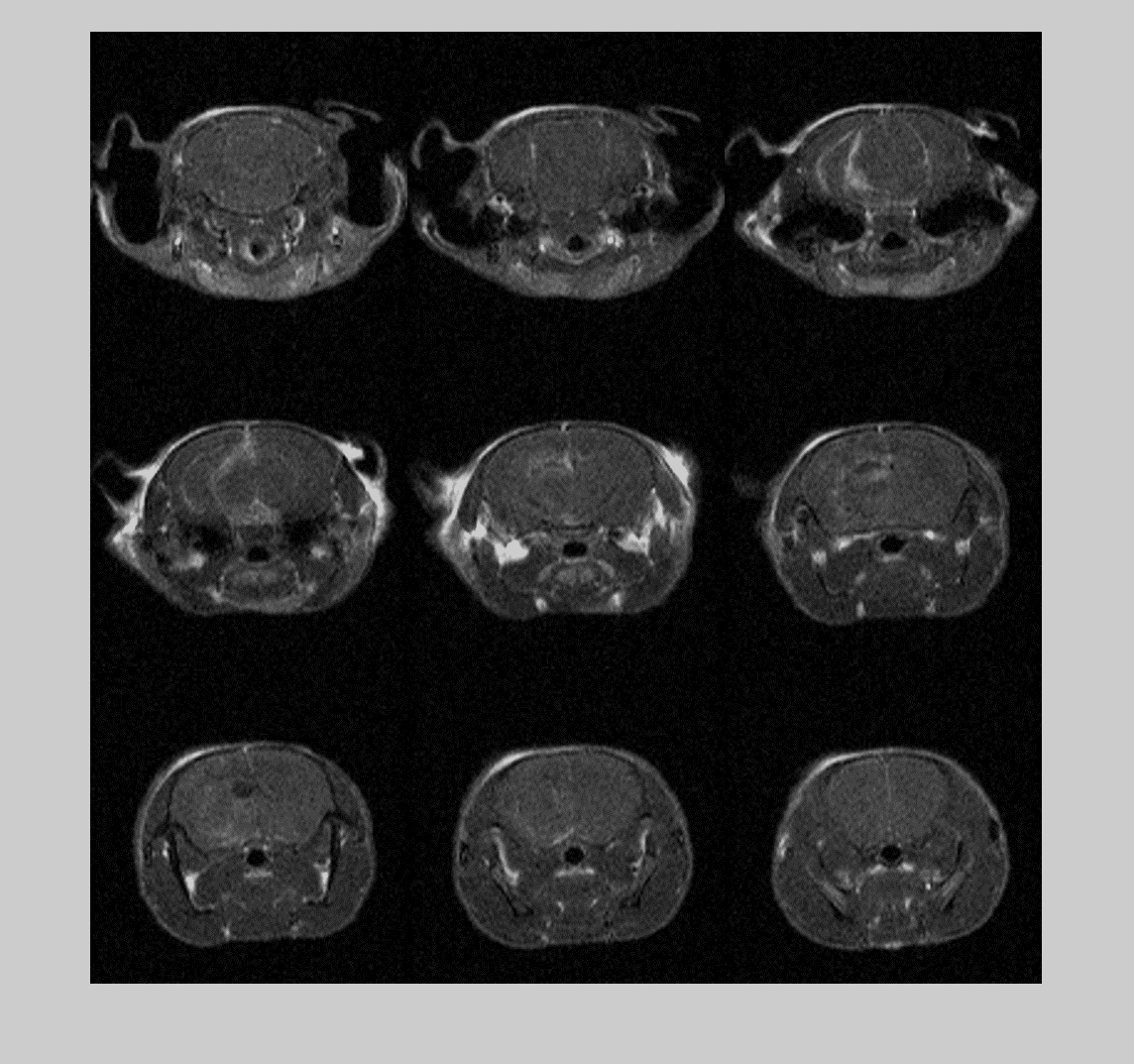

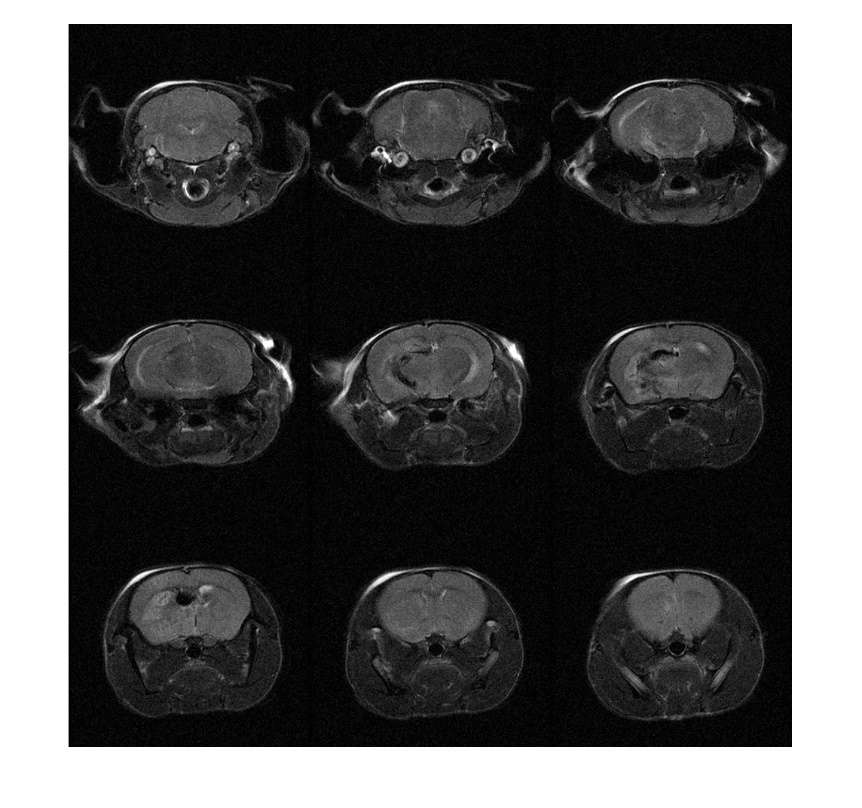


Mouse 5

T1 T2


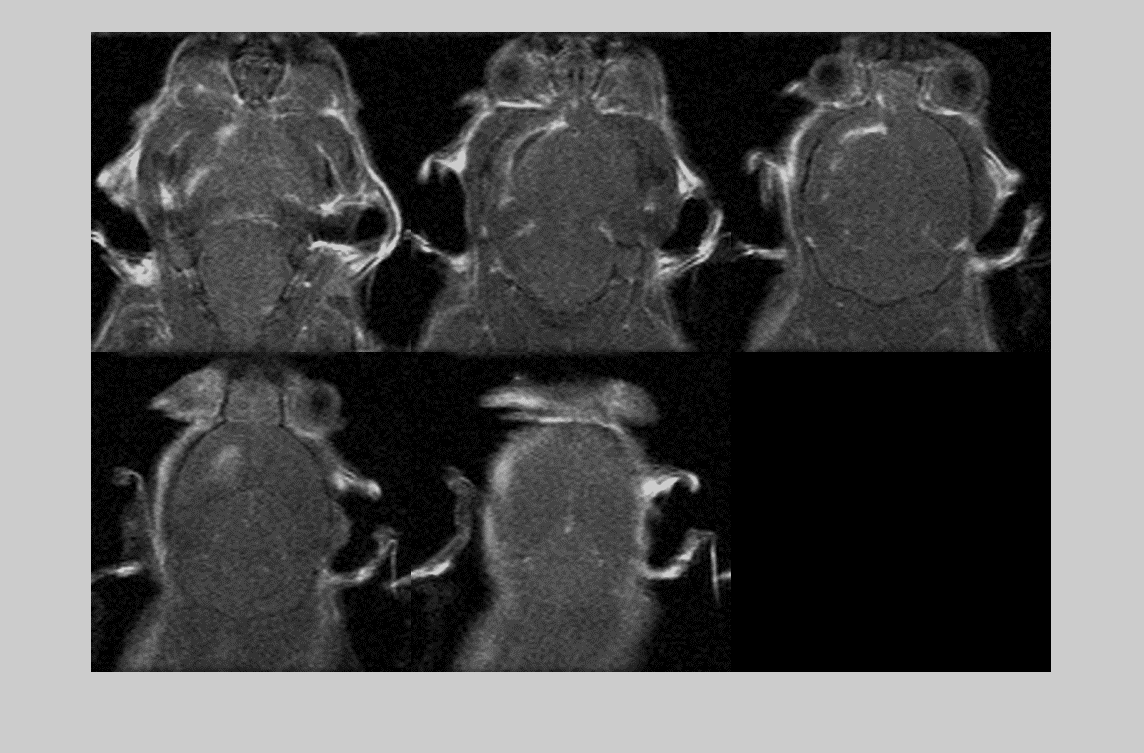

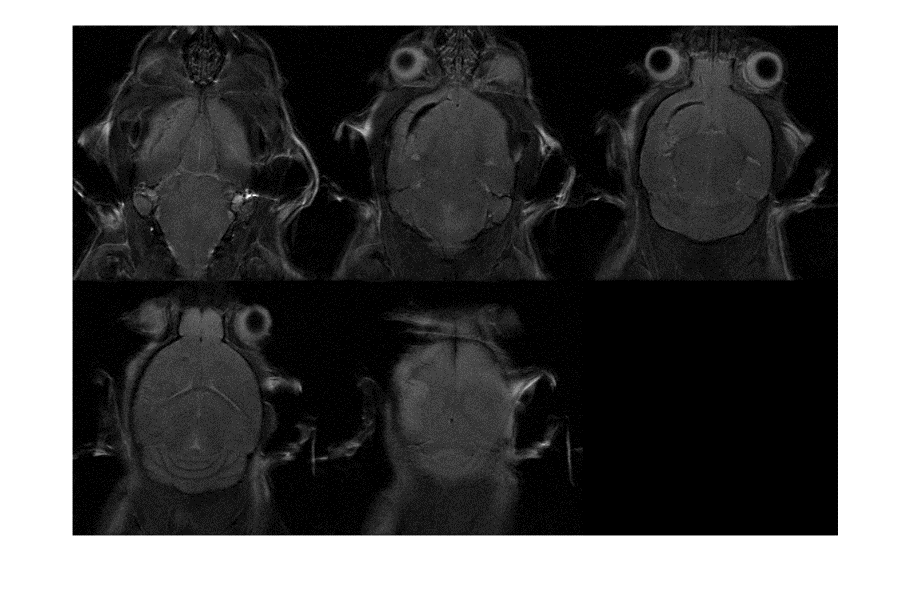


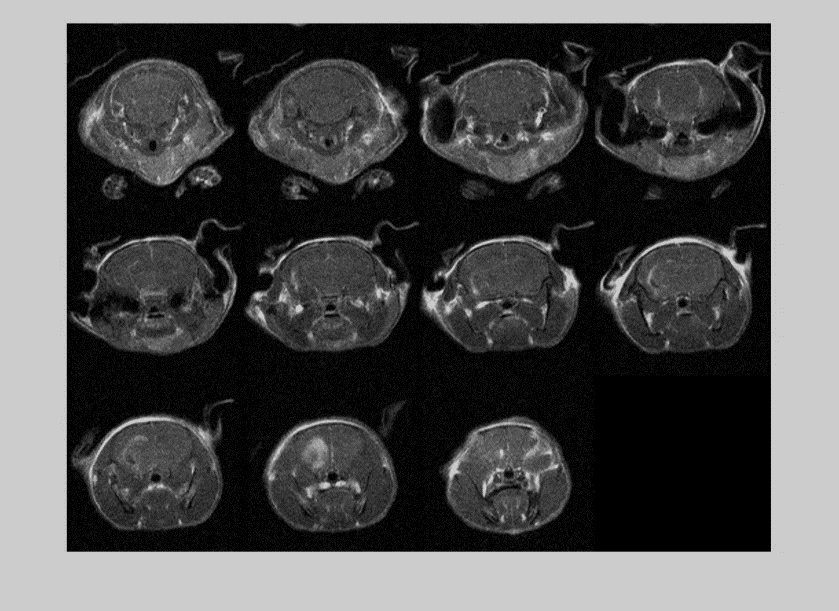

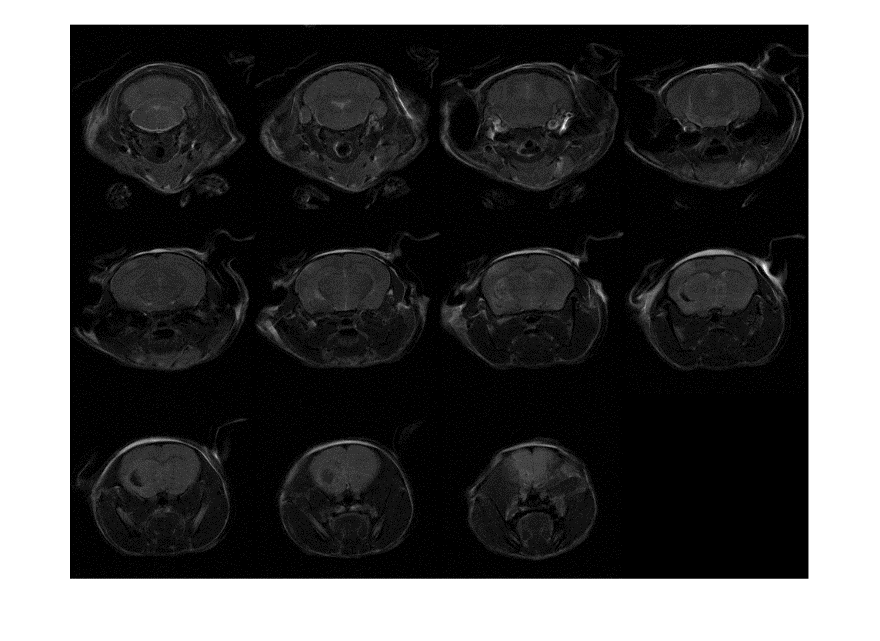


Mouse 6

T1 T2


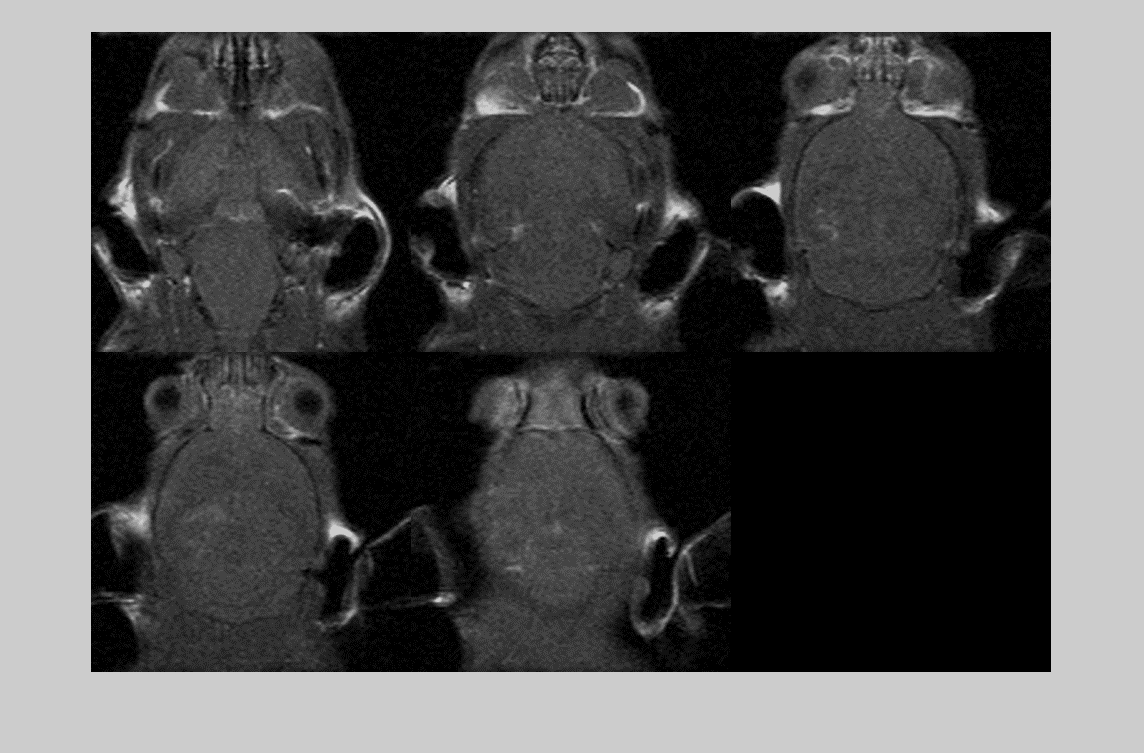

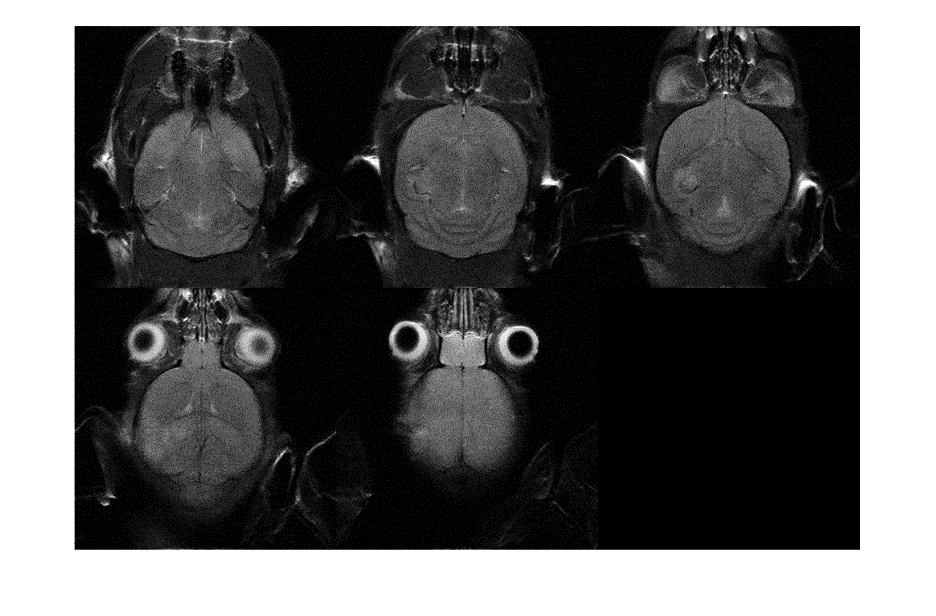


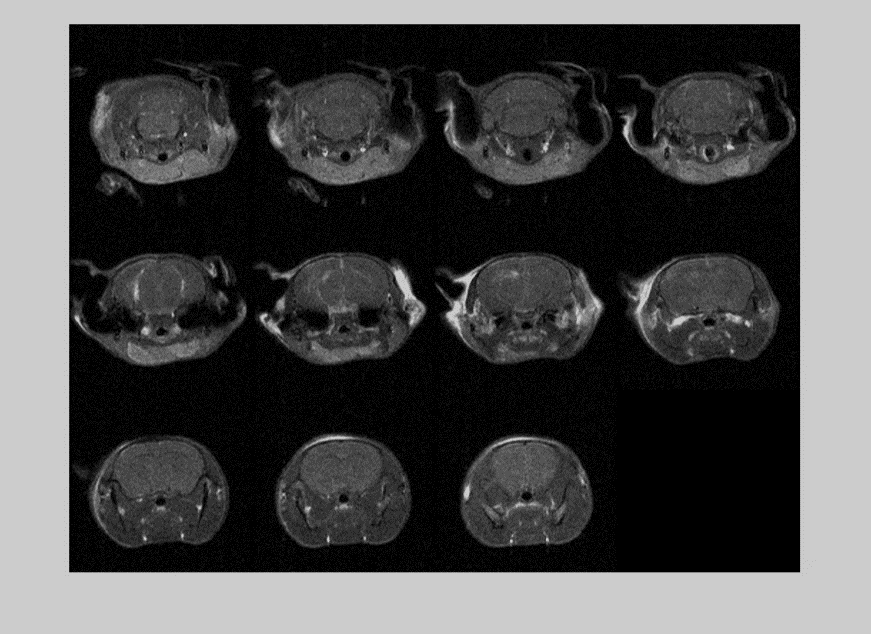

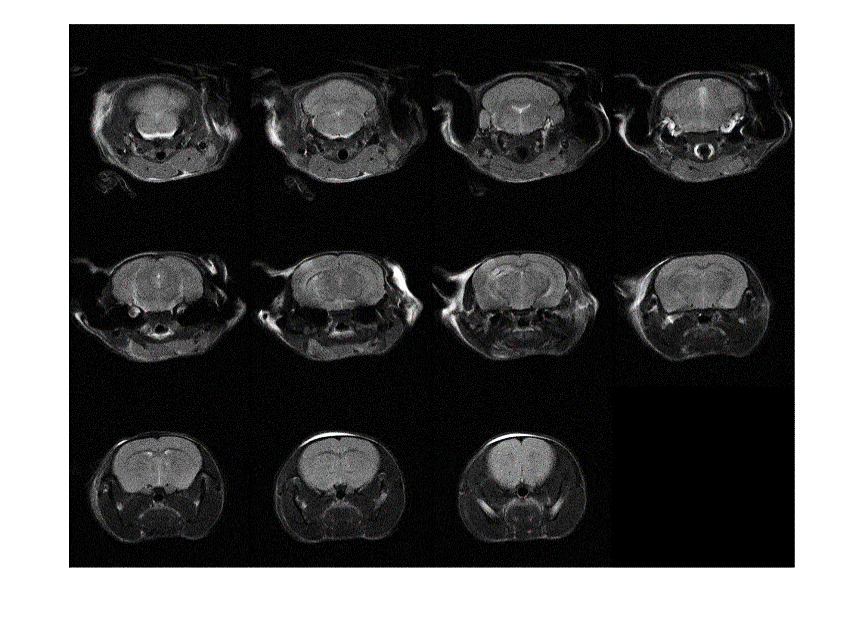


Mouse 7

T1 T2


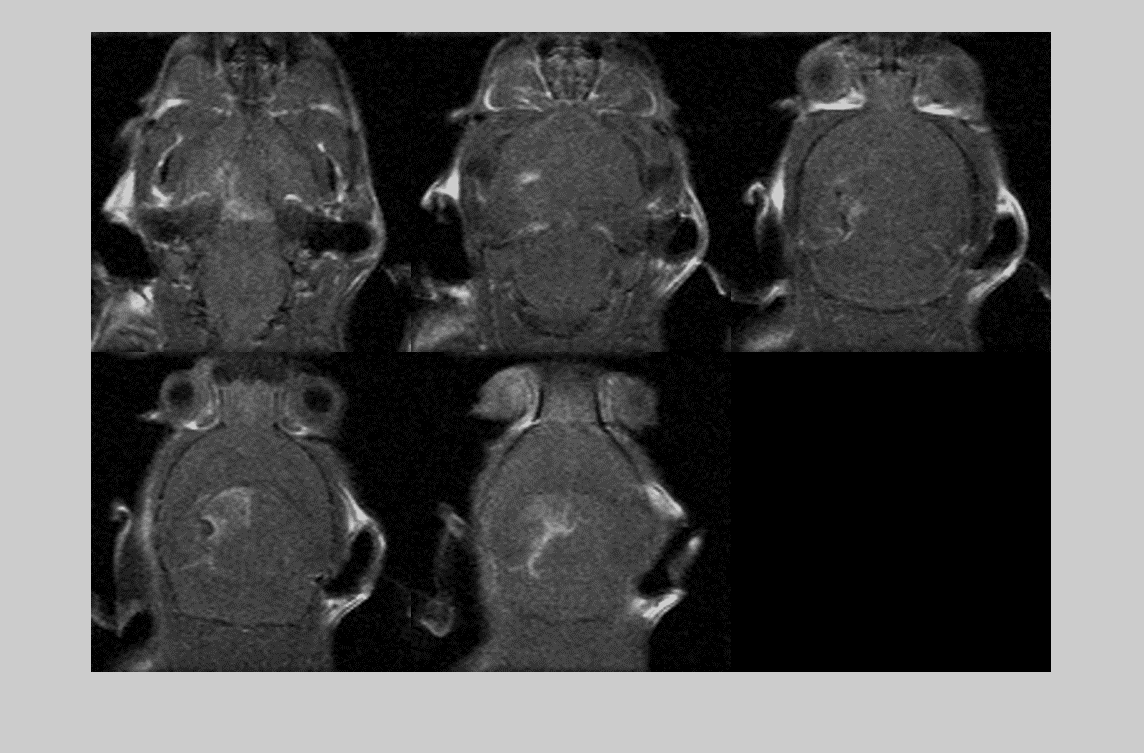

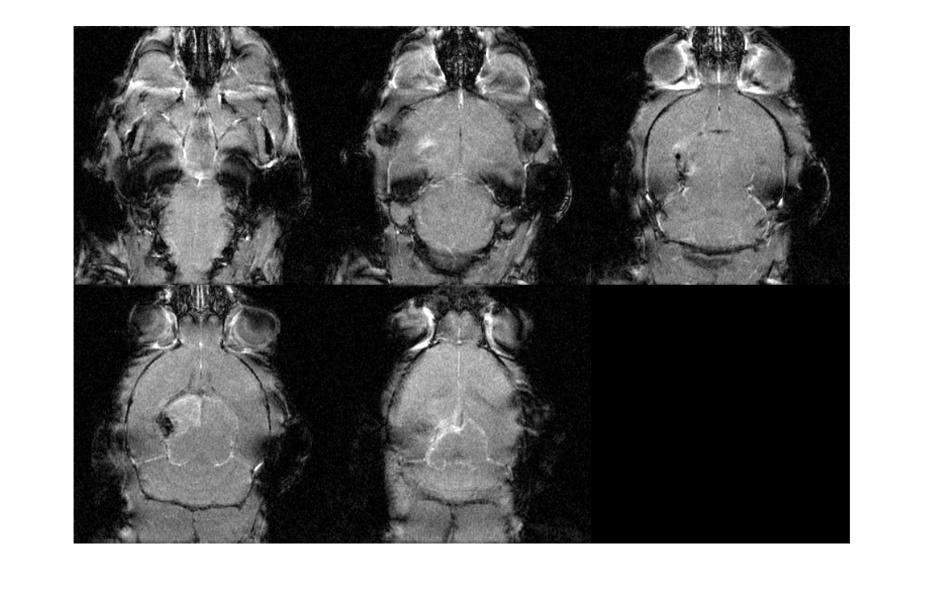


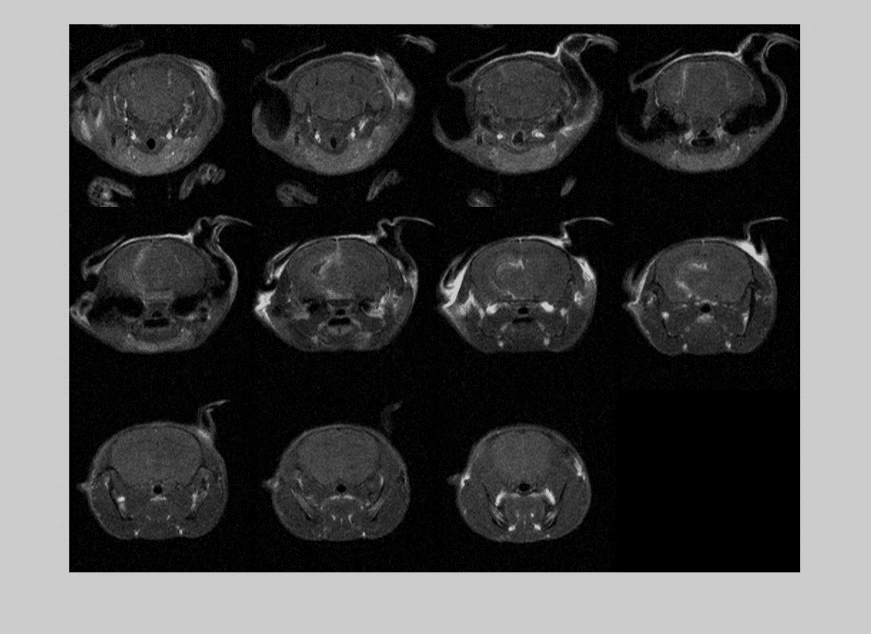

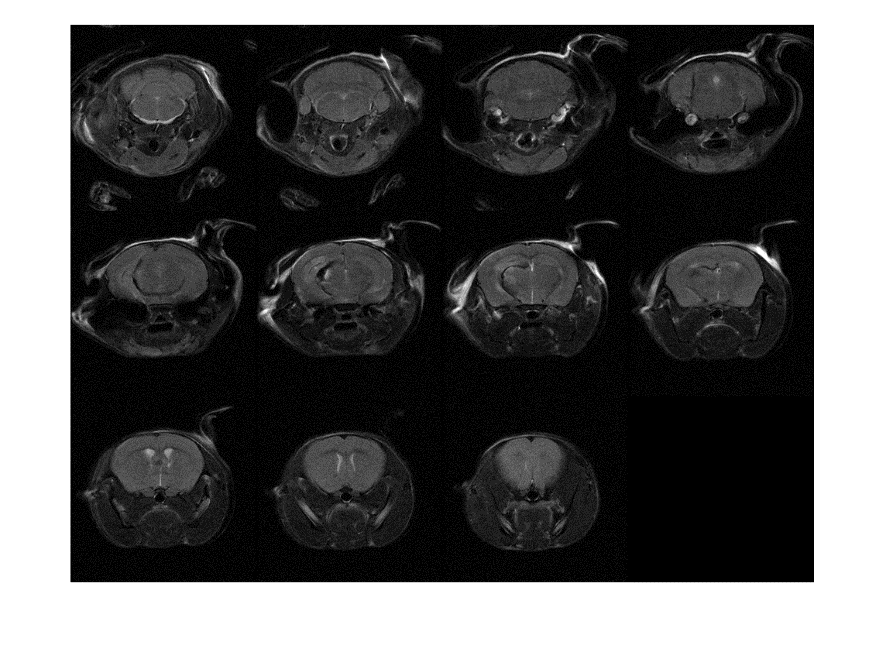

Supplement: Fig. S1 — T1 weighted and T2 weighted images (coronal and axial slices) of all animals. [file mmc1.docx]

Mouse 1


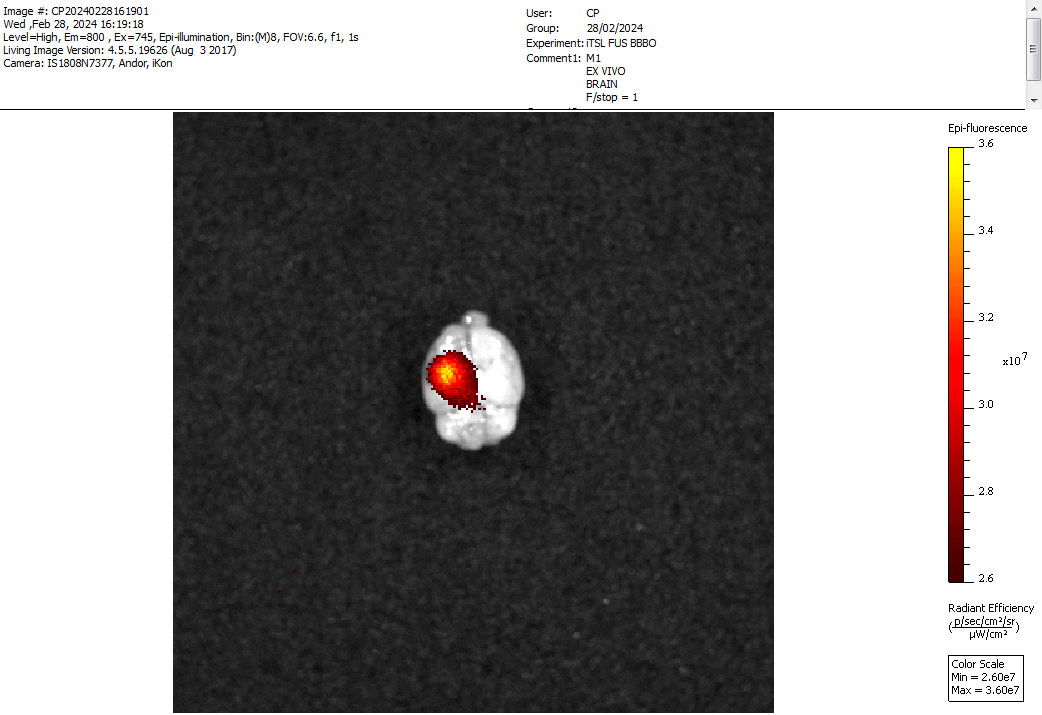


Mouse 2
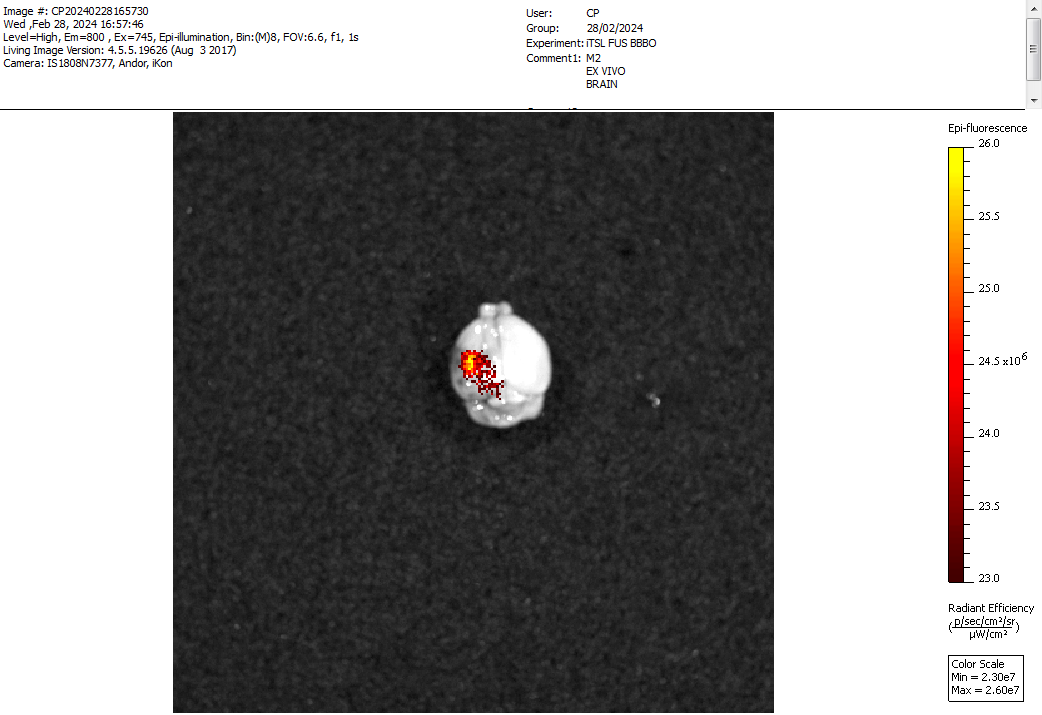


Mouse 3


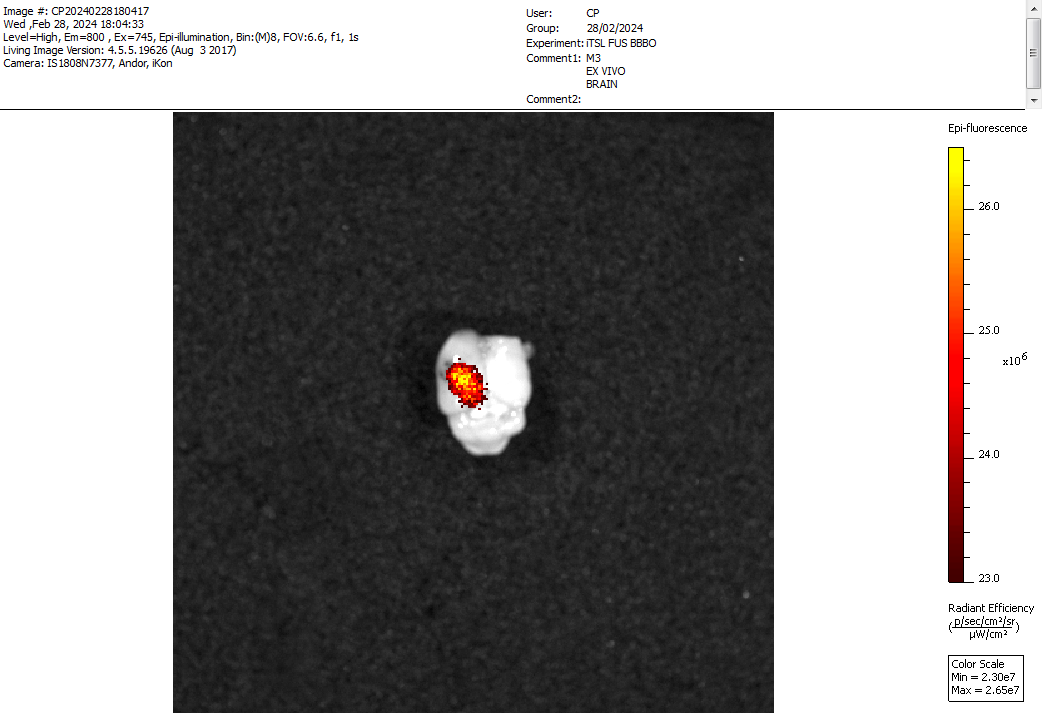


Mouse 4


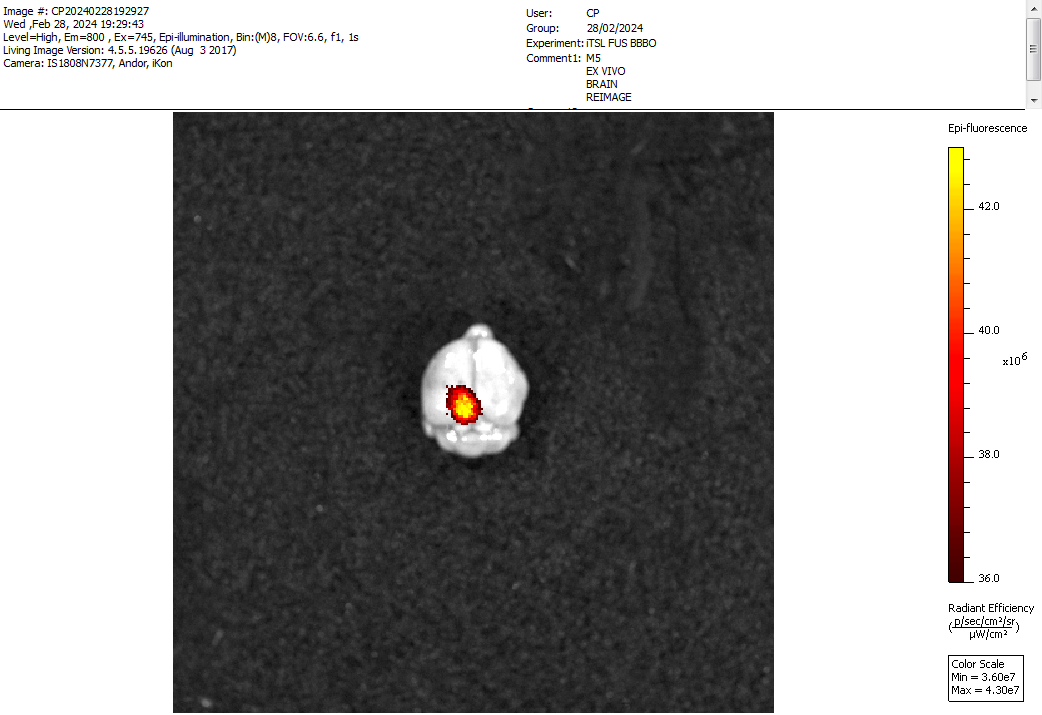


Mouse 5


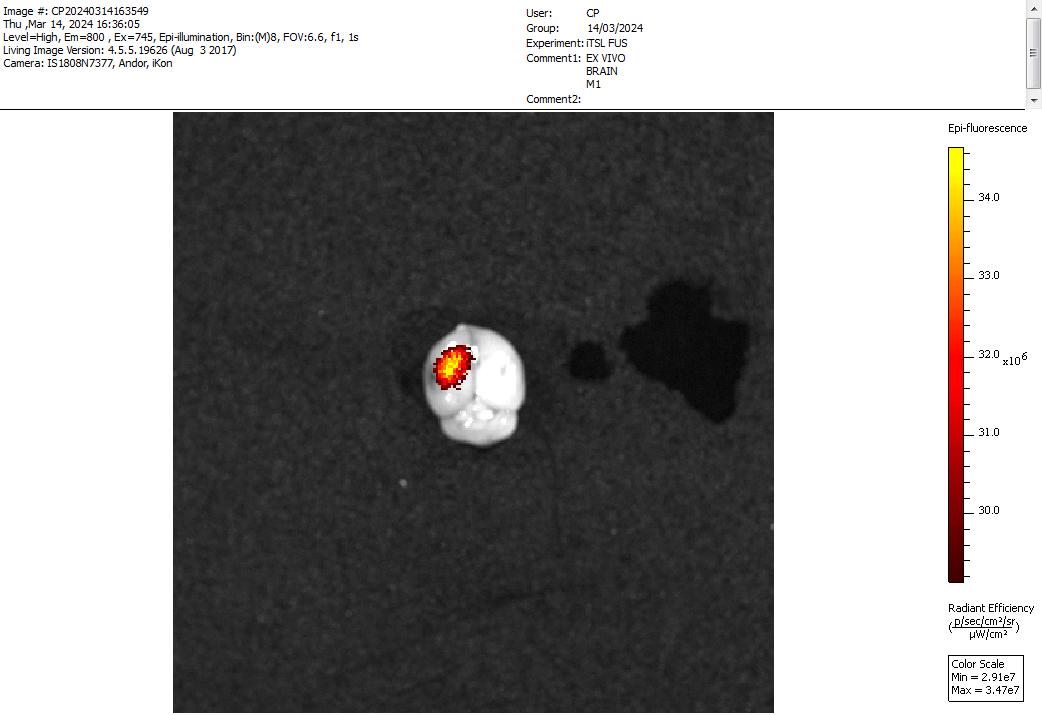


Mouse 6


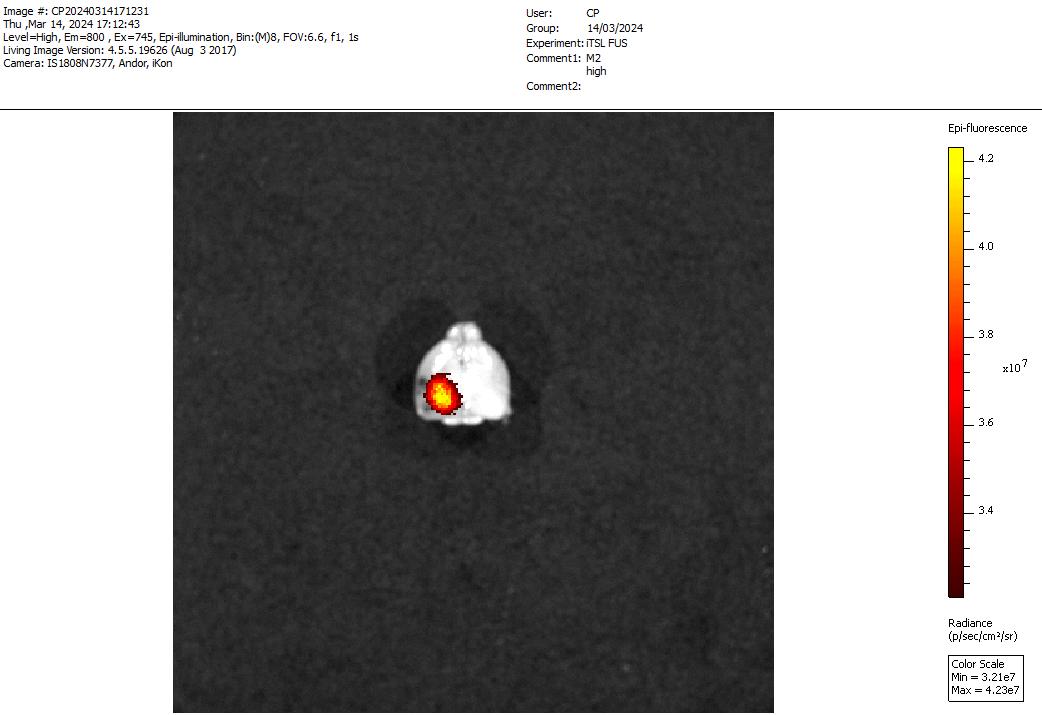


Mouse 7


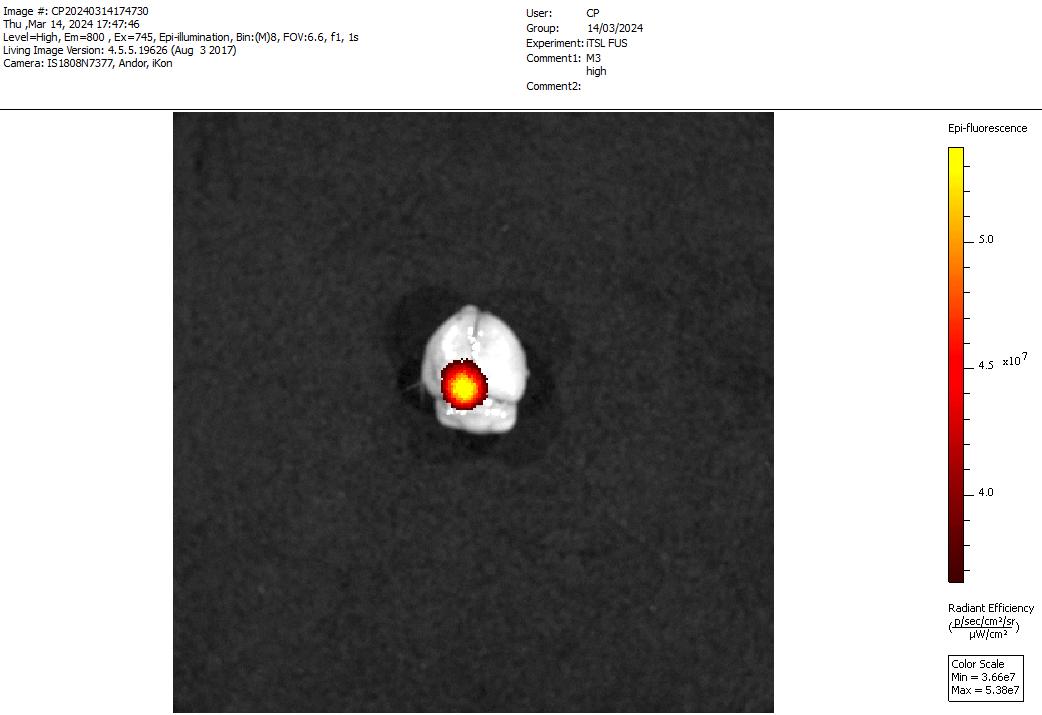

Supplement: Fig. S2 — Ex vivo whole brain NIRF images. [file mmc2.docx]
